# SMARCC2 mediates the regulation of DKK1 by the transcription factor EGR1 through chromatin remodeling to reduce the proliferative capacity of glioblastoma

**DOI:** 10.1038/s41419-022-05439-8

**Published:** 2022-11-23

**Authors:** Chiyang Li, Tong Wang, Junwei Gu, Songtao Qi, Junjie Li, Lei Chen, Hang Wu, Linyong Shi, Chong Song, Hong Li, Liwen Zhu, Yuntao Lu, Qiang Zhou

**Affiliations:** 1 Department of Neurosurgery, Nanfang Hospital, Southern Medical University, Guangzhou, China; 2 Nanfang Neurology Research Institution, Nanfang Hospital, Southern Medical University, Guangzhou, China; 3 Nanfang Glioma Center, Guangzhou, China; 4 Department of Hematology, Nanfang Hospital, Southern Medical University, 510000 Guangzhou, Guangdong P.R. China

**Keywords:** CNS cancer, Chromosomes

## Abstract

Switch/sucrose-nonfermenting (SWI/SNF) complexes play a key role in chromatin remodeling. Recent studies have found that SMARCC2, as the core subunit of the fundamental module of the complex, plays a key role in its early assembly. In this study, we found a unique function of SMARCC2 in inhibiting the progression of glioblastoma by targeting the DKK1 signaling axis. Low expression of SMARCC2 is found in malignant glioblastoma (GBM) compared with low-grade gliomas. SMARCC2 knockout promoted the proliferation of glioblastoma cells, while its overexpression showed the opposite effect. Mechanistically, SMARCC2 negatively regulates transcription by dynamically regulating the chromatin structure and closing the promoter region of the target gene DKK1, which can be bound by the transcription factor EGR1. DKK1 knockdown significantly reduced the proliferation of glioblastoma cell lines by inhibiting the PI3K–AKT pathway. We also studied the functions of the SWIRM and SANT domains of SMARCC2 and found that the SWIRM domain plays a more important role in the complete chromatin remodeling function of SMARCC2. In addition, in vivo studies confirmed that overexpression of SMARCC2 could significantly inhibit the size of intracranial gliomas in situ in nude mice. Overall, this study shows that SMARCC2, as a tumor suppressor, inhibits the proliferation of glioblastoma by targeting the transcription of the oncogene DKK1 through chromatin remodeling, indicating that SMARCC2 is a potentially attractive therapeutic target in glioblastoma.

## Introduction

Grade IV glioblastoma (GBM) is the most common primary brain cancer in adults [1]. Less than 10% of patients achieve 5-year survival under the current therapeutic regimen [2]. Surgery supplemented by postoperative radiotherapy, chemotherapy, synchronous and adjuvant temozolomide (TMZ), tumor treating fields (TTFs), or other treatments is still the main therapeutic approach for managing glioma [3]. However, regardless of the treatment strategy used, the median survival time is short, mainly due to tumor recurrence and resistance to treatment [4]. The key factors of resistance to treatment include the heterogeneity of these tumors, the genomic deletion of tumor suppressor genes, the diffuse and osmotic growth of tumors and the existence of the blood–brain barrier. Deletion of tumor suppressor genes plays an important role [5].

The switch/sucrose-nonfermenting (SWI/SNF) complex is a member of the chromatin remodeling protein family [6]. To date, 29 components have been identified to be involved in the assembly of three different SWI/SNF complexes, including classical BRG1/BRM-related factor (CBAF), polybromine-related BAF (PBAF), and the recently reported noncanonical BAF (ncBAF) complex [7]. As the prototypical chromatin remodeling complex, SWI/SNF has nucleosome sliding activity and unique ejection activity, producing nucleosome deletion regions (NDRs), which are very important for transcriptional regulation [8]. Therefore, SWI/SNF plays an important role in fundamental cellular processes such as gene expression, DNA repair and replication and the regulation of high-order chromatin organization. Mutation and absence of the expression of the SWI/SNF core protein are found in more than 20% of different tumors, such as esophageal adenocarcinomas, lung cancers, ovarian clear cell carcinomas, and endometrioid carcinomas, making these complexes the most common altered targets in human carcinoma [9]. However, little is known about these complexes in glioma, and their potential tumor inhibition mechanism is not fully understood. In most cases, they are loss-of-function (LOF) mutations, resulting in the deletion of mutant subunits at the protein level [10]. However, identifying the mechanism of SWI/SNF mutation in promoting cancer remains a challenge.

SMARCC2 (BAF170) is one of the constant core subunits of the ATP-dependent chromatin-remodeling BAF (BRG1-related factor) complex [11]. As the scaffold of the basic module, the SMARCC2 subunit combines all other basic subunits and passes through the complex head, thumb, palm and finger submodules, playing a key role in the early stage of complex assembly [12]. SMARCC2/SMARCC1 double knockout mice showed proteasome-mediated degradation of the whole BAF complex, resulting in damage to the overall epigenetic and gene expression programming of forebrain development [13]. SMARCC has also been reported as one of the chromatin remodeling genes involved in autism spectrum disorder (ASD). Despite its important biological role [14], only a small number of studies have related it to human glioblastoma. SMARCC2 contains two functional domains: SWIRM (Ile424–Thr521) and SANT (Ser596–Pro647). The SANT domain, the preHSA domain of SMARCC4, and the C-terminal helix of SMARCD1 together compose the thumb part of the basic module of the SWI/SNF complex, while the SWIRM domain, two RPT domains, and one C-terminal of the SMARCB1 α helix (αC), the Req domain of TDP2 and an insert derived from ARID1A (ARID1A insert) form the complex [15]. The head directly binds to the histone octamer at the bottom, whose relationship also reflects that the SWIRM domain may play a more key role in chromatin remodeling than the SANT domain. On the other hand, a region of SMARCB1 (AA 169–385, SMARCB1 (169–385)) and the SWIRM domain of SMARCC2 (AA 423–518, SMARCC2 (423–518)) form a stabilizer complex, and the inactivation of SMARCB1 has been reported in almost all malignant rhabdoid tumors. Studies have shown that the assembly of SMARCB1–SMARCC2 subcomplexes is very important for tumor suppression [16]. When SMARCC2 disease-related mutations inhibit the formation of SMARCB1–SMARCC2 subcomplexes, tumorigenesis eventually occurs. Therefore, this study focuses on the possible molecular mechanism of SMARCC2 in the occurrence and development of glioblastoma. In this study, we found that SMARCC2 can stabilize the overall structure of SWI/SNF in glioblastoma cell lines. It can also inhibit tumor development by mediating the expression of the transcription factor EGR1 through chromatin remodeling and then inhibiting the activation of the PI3K-ATK pathway. In addition, through bioinformatics analysis of The Cancer Genome Atlas (TCGA) data, it was found that the levels of SMARCC2 and DKK1 were closely related to the prognosis of GBM patients. Mutation analysis of the selected glioblastoma patients found that SMARCC2 was mutated in up to 20% of cases. In summary, our findings elucidate the possible mechanism of GBM tumor development.

## Methods

### Cell line and culture

Human GBM cell lines (U87MG, LN229, T98G, A172, and U118MG) were purchased from the American Type Culture Collection (ATCC, Manassas, VA). U251 cells were obtained from Shanghai Institutes for Biological Sciences, Chinese Academy of Sciences (Beijing, China). Cells were cultured in DMEM (containing 4.5 g/L glucose, Gibco 11995065) with 10% FBS (Gibco, 16140071), 100 U/ml penicillin and 100 µg/ml streptomycin in a humidified atmosphere at 37 °C with 5% CO_2_. Short tandem repeat loci and Amelogenin loci genotyping of this cell line was authenticated in January 2014. All the cell lines have been tested and shown to be no mycoplasma contamination.

### Generation of genome-edited SMARCC2 knockout clones

CRISPR guides (single-guide RNA) were designed against Exon 1 of SMARCC2 (no. NM_003075). The sequence of the guide was “GGCCTCGTAGTACTTCACGT”. The first base of the sequence was removed, and “TGG” and “GTTTT” bases are added at the end as oligo F (GCCTCGTAGTACTTCACGTTGGGTTTT), which is inversely complementary to oligo F, and “CGGTG” was added at the tail to form oligo R (CCAACGTGAAGTACTACGAGGCCGGTG). The oligonucleotide (Sigma-Aldrich) was subcloned into the p2U6-Cas9 plasmid vector [17]. Then, the CRISPR–Cas9 plasmid was transfected into cell suspensions of the glioblastoma cell line U118MG (2 million cells) by electrotransfection (5 ms, 200 V, two pulses). After electrotransfection, the cell suspensions were transferred into 10-cm cell culture dishes, and DMEM cell culture medium containing 10% FBS was added. The culture medium was changed after 6–8 h, when the cells had adhered to the walls of the dish.

### Single-cell clone culture

In this study, the glioblastoma cell line U118MG with CRISPR–Cas9-specific knockout of SMARCC2 was used for single-cell clonal culture. The U118MG cell line, electrotransfected with sgRNA-SMARCC2 plasmid in the exponential growth stage, was trypsin-digested to obtain a cell suspension. Then, the cell suspension was counted and continuously diluted to ten cells/ml with conditioned medium. Then, the suspension was assembled into a 96-well plate at a concentration of ten cells per well (Corning Inc., NY, USA). The cells are arranged randomly and obey the Poisson distribution. Some culture wells had no cells, some culture wells had one cell, and the rest had two or more cells. Single-cell culture medium is in high glucose DMEM medium containing 10% fetal bovine serum supplemented with N2 nerve growth factor. After 12 h of incubation, the plates were examined under a phase contrast microscope at ×100 magnification. The wells with only a single living cell were marked with a diamond. During subsequent incubation in 96-well plates for ~30 days, during which the labeled wells are maintained by changing the conditioned medium every 4–5 days. When clones of 100–200 cells appeared in the labeled wells, they were transferred to a six-well plate for further division, and the slower-growing clones were returned to the incubator undisturbed until they reached the desired density. Clones transferred to six-well dishes expanded to ~80–90% confluence after ~6 weeks and were further subdivided into 100-mm dishes for further analysis.

### DKK1 and EGR1 knockdown array

SiRNAs targeting DKK1 and EGR1 were provided by RiboBio (Guangzhou, China). Twenty-four hours after inoculation, the cells were transfected with Lipofectamine 2000 reagent (Invitrogen, 11668-019; Carlsbad, CA, USA). The transfection complex was prepared according to the manufacturer’s instructions and added directly to the cells at a final oligonucleotide concentration of 100 nM. The transfection medium was changed 6–8 h after transfection. The transfection efficiency was evaluated 72 h after transfection using qRT–PCR and western blotting.

### SMARCC2 overexpression and knockout array

Cells were prepared and infected with control or SMARCC2-overexpressing LVs (GenePharma, Suzhou; SMARCC2 NCBI reference sequence: NM_003075). SMARCC2-overexpressing LVs (oeSMARCC2), adenovirus and DMEM cell culture medium containing 10% were mixed to a concentration of 100 nM and directly added to U87MG cells. The cell culture medium was changed after 12 h, and the transfection efficiency was evaluated by qRT–PCR and western blotting after 48 h. After the electric transfection of the CRISPR–Cas9-SMARCC2 (sgSMARCC2) plasmid, the cells were further monoclonally cultured. U118MG cells with successful transfection and complete knockdown of SMARCC2 were selected for qRT–PCR and western blotting.

### RNA isolation and qRT–PCR array

According to the reagent instructions, total RNA was extracted from cultured tumor cells using RNAiso Plus* (TaKaRa, 9109; Shiga, Japan). A PrimeScript™ RT kit and gDNA Eraser (Takara, rr047a) paired with 1 μg of total RNA was used for each sample in a 20 µL reaction system to synthesize cDNA. Then, 1 μL of cDNA library was added to the 10 μL LPCR mixture. TB Green® *Premix Ex* Taq™ (TLI RNaseH Plus) (Takara, rr420q) was used to determine the threshold cycle (Ct) value of each sample in a Quant Studio 5 real-time PCR system (Thermo Fisher Scientific). GAPDH was used as a reference gene in these studies. The quantitative cycle number was calculated according to the following formula (ΔΔ difference between CT) and relative quantification (RQ): Δ CT (sample) = CT (target) − CT (reference), ΔΔ Ct = Δ CT (sample) − Δ CT (calibrator), RQ = 2 − ΔΔ Ct. Each independent experiment was conducted in triplicate. For a detailed list of primers, refer to the Supplementary information.

### Western blot analysis and antibody

The cell samples were lysed in RIPA buffer (Sigma, r0278), and the protein concentration was determined using a BCA protein detection kit (Solarbio Life Sciences, pc0020; Beijing, China). Total protein was measured on an SDS–PAGE gel by 8–15% (20 µl volume contained 30–50 μg) and transferred to PVDF membranes (Millipore, IPVH00010; Billerica, MA, USA). The membrane was then blocked with 5% skimmed milk (BD Biosciences, 232100; San Jose, CA, USA) or 5% BSA (Solarbio Life Sciences, a8020) in TBS containing 0.1% Tween-20 (Sigma, P9416), incubated with primary antibody at 4 °C overnight (Rat anti-SMARCC2 (Cell Signaling Technology, 12760, 1:1000), rat anti-SMARCC1 (Cell Signaling Technology, 11956, 1:1000), anti- SMARCA4 (Cell Signaling Technology, 49360, 1:1000), anti- SMARCB1 (Cell Signaling Technology, 91735, 1:1000), rat anti-DKK1 (Cell Signaling Technology, 48367, 1:1000), rabbit anti-EGR1 (Cell Signaling Technology, 4154, 1:1000), anti- Akt (Cell Signaling Technology, 4691, 1:1000), anti-p-Akt (Cell Signaling Technology, 4060, 1:1000), anti- PI3K (Cell Signaling Technology, 4257, 1:1000), anti-p-PI3K (Cell Signaling Technology, 17366, 1:1000), anti-GAPDH (Cell Signaling Technology, 5174, 1:1000) and mouse anti-β-actin (Cell Signaling Technology, 3700, 1:1000)), and then incubated with HRP-conjugated rabbit (Cell Signaling Technology, 7074, 1:2000) or mouse secondary antibody (Cell Signaling Technology, 7076, 1:2000) at room temperature for 1–2 h. The band intensity was quantified using a Tanon-5500 chemiluminescence imaging system (Tanon Science & Technology; Shanghai, China). The chemiluminescence substrate was an Immobilon ECL Ultra Western HRP substrate (Millipore, WBULS0500). GAPDH or β-actin was used as a loading control.

### Cell viability assay

Cell proliferation was assessed using Cell Counting Kit-8. First, cells were plated in 96-well plates at a density of 5000 cells per well. After overnight incubation, cell viability was measured using Cell Counting Kit (CCK)-8 (Beyotime, Shanghai, China). Briefly, 10 μL of CCK-8 reagent was added to each well, and the cells were incubated with the reagent for 2 h. Then, the absorbance at 450 nm was measured by a microplate reader (Bio-Rad).

### Colony-forming cell assay

Cells (200 cells/well) were seeded onto six-well culture plates and cultured in DMEM supplemented with 10% FBS. The cells were incubated for 10–14 days at 37 °C and 5% CO_2_. Colonies were then stained with 0.1% crystal violet (Sigma-Aldrich) and counted. For each set of clones, three independent assays were carried out.

### EdU staining

The proliferation rate of U87MG and U118MG cells was evaluated by EdU (5-ethyl-2’-deoxyuridine, Life Technologies) labeling. Briefly, 1 × 10^6^ U87MG or U118MG cells were inoculated into 6-cm tissue culture dishes and cultured in a cell incubator at 37 °C for 12 h. EdU was then added to the medium at a final concentration of 10 μM. Then, the cells were cultured for another 2 h, following the manufacturer’s instructions. Images were captured with a fluorescence microscope.

### RNA sequencing

RNA-Seq was performed on sgNC and sgSMARCC22 (U118MG cell lines) as well as oeNC and oeSMARCC22 (U87MG cell lines). Total RNA was extracted from each group by using the TRIzol reagent (Thermo Fisher). The quality of the RNA samples was evaluated using Nanodrop 2000 (Thermo Fisher) and Bioanalyzer 2100 (Agilent) instruments [18]. The RNA-Seq library was constructed using a TruSeq Stranded mRNA library preparation kit (Illumina) and sequenced with HiSeq X Ten (Illumina) in a biomarker (DeiJing, China) under the PE150 protocol. These readings were aligned with human reference genome NCBI build 39 (GRCh39), and the reads per kilobase per million mapping reads (RPKM) were calculated. DESeq2 provides statistical routines for determining differential expression in digital gene expression data using a model based on the negative binomial distribution. The resulting *P* values were adjusted using the Benjamini–Hochberg approach for controlling the false discovery rate. Genes with an adjusted *P* value < 0.01 found by DESeq2 were assigned as differentially expressed and plotted in a heatmap or volcano map with ggplot (R software).

### ATAC-seq sequencing

ATAC-Seq was performed for sgNC and sgSMARCC2 (U118MG cell line) as well as oeNC and oeSMARCC2 (U87MG cell line). A total of 50,000 cells were centrifuged at 4 °C for 5 min at 500 × *g*, and then the supernatant was removed. The cells were washed with cold PBS once. After centrifugation at 4 °C for 5 min at 500 × *g*, the supernatant was removed. The cells were then suspended in cold lysis buffer. After centrifugation at 4 °C for 10 min at 500 × *g*, the supernatant was removed. The transposase reaction system was configured with Tn5 transposase. The cell nuclei were suspended in the transposase reaction system, and the DNA was purified after incubation at 37 °C for 30 min. The PCR system was configured with the purified DNA, and then the PCR amplification reaction was performed. After DNA purification, the final DNA libraries were sequenced on the Illumina platform [19].

### ATAC-seq data analysis

#### Illumina sequenced read processing

Raw reads were filtered with Cutadapt software to remove adapters, reads less than 35 bp in length and low-quality reads (including reads with an N ratio greater than 10% and reads with a base quality value Q ≤ 10 accounting for more than 50% of the entire read). The high-quality clean reads provided in FASTQ format were used for subsequent analysis.

#### Mapping to the reference genome

Bowtie2 software was used to compare the high-quality reads obtained from the sequencing of each sample with the reference genome to obtain the alignment efficiency of the sample reads and the position information of the reads on the genome. DeepTools v2.07 was used to map the density distribution of sequencing reads in the 3 kb intervals upstream and downstream of the TSS of each gene, and the results are presented as heatmaps.

#### Sample correlation

The read abundance in the whole genome was statistically analyzed by the sliding window method, taking 10 kb as the unit length of the interval and dividing chromosomes into multiple small windows. The number of mapped reads in each window was counted as the read abundance, and the Pearson correlation coefficient of the normalized read abundance was calculated. A sample correlation clustering heatmap was made.

#### Detection of the genome-wide peak region

MACS2 v2.1.1 software was used to perform peak extraction.

### Protein structure visualization analysis

The PDB file of the 3D structure of SMARCC2 (PDB ID: 6LTJ, resolution: 3.70 Å, method: electron microscopy) was obtained from the RCSB PDB database (https://www.rcsb.org/) and imported into PyMOL software (v1. 7.4.5 version) [20]. Each fragment was selected according to the amino acid sequence (Table 1), different fragments were marked with different colors, and then the position of each fragment in the spatial structure was observed. To analyze the interaction between SWIRM (SMARCC2B) and RPT2 (SMARCB1), SWIRM (SMARCC2B) and RPT2 (SMARCB1) were selected in PyMOL software, and find→polar contacts→to any atom for SWIRM (SMARCC2B) were used to analyze its relationship with RPT2 (SMARCB1) with interacting amino acid residues. The results were finally visualized in PyMOL software.

**Table 1:** Amino acid sequence corresponding to each fragment.

Amino acid sequence	Fragment
424-515	SWIRM (SMARCC2B)
602-641	SANT (SMARCC2B)
259-356	RPT1 (SMARCB1)
184-249	RPT2 (SMARCB1)
358-378	αC (SMARCB1)
15-109	H2A
31-120	H2B
18-136	DNA

Amino acid fragments corresponding to different functional structures of SMARCC2/SMARCB1 and H2A/H2B, and the DNA fragments bound to protein subunits are also shown in the table.

### Luciferase reporter assay

U87MG and U118MG cells were cotransfected with pRL-TK, DKK1-Promo plasmid, and EGR1 plasmid. Forty-eight hours after transfection, luciferase activity was determined using a Dual-Luciferase Reporter Assay Kit (Promega, E1910; Madison, WI, USA) and a BMG microplate reader (BMG LabTech, Clariostar; Cary, NC, USA) according to the manufacturer’s instructions. To reduce the luciferase level variability caused by the different efficiencies of plasmid transfection into cells, the luciferase level of pRL-TK was selected as the control. All experiments were performed in triplicate and repeated at least three times.

### Histological evaluation and immunohistochemical staining

The whole skull of each tumorous nude mouse was collected, fixed in 4% paraformaldehyde for 24–48 h, embedded in paraffin, cut into continuous 4-µm thick sections, and stained with hematoxylin and eosin (LEAGENE, DH0006-2 Beijing, China). Immunohistochemical staining was performed using a ZSGB-BIO PV-9000 kit (Beijing, China) according to the manufacturer’s instructions. Tissue sections from paraffin-embedded human GBM specimens and xenograft tissues were stained with specific antibodies (refer to Supplementary information) or nonspecific IgG as negative controls.

### In vivo xenografts

GBM cells with negative control or SMARCC2 (oe) or SMARCC2 (ko) (1 × 10^6^) were intracranially injected into 4-week-old female athymic nude mice (10 μL PBS per mouse). Each group of experimental animals was blinded. The frontal cortex was injected (coordinates are as follows: *x* = 2.5, *y* = −3.5, *z* = −2.5, anterior fontanelle as coordinate point 0 of X and y). Four weeks after injection, the tumor was confirmed by MRI (Bruker Medical Inc., Billerica, MA, USA), and then the animals were killed under abdominal anesthesia with sodium pentobarbital at the end stage. The brain was then harvested after cardiac perfusion with heparin saline and 4% PFA. Tumor volume was calculated using the following formula: volume (mm^3^) = 4/3 × 3.14 × radius (mm)^3^.

### Data collection

We obtained processed RNA sequencing and clinicopathological data from TCGA (The Cancer Genome Atlas Program - NCI), including 166 GBM samples and 530 LGG samples. We used Levene’s test and Wilcoxon test of R3.6.1 to analyze SMARCC2 and DKK1 expression differences in LGG and GBM samples (*P* < 0.05). When it comes to survival analysis, we filtered out individuals with no survival information. CBioPortal (http://www.cbioportal.org/) provided information of gene point mutation and copy number variation(CNA), which we combined to show the heatmap of SMARCC2 alteration in glioma.

### Statistical analysis

All experiments were conducted in triplicate. The mean and standard error of the mean were reported as appropriate. Statistical analysis was performed using Prism (GraphPad, San Diego, CA). Analysis of variance (ANOVA) was performed for multiple group comparisons, followed by post hoc Dunnett’s test (group compared with a control group) or post hoc Tukey’s test (to determine the differences between subgroups). An unpaired two-tailed Student’s *t* test was used for comparison. The survival curve was estimated by the Kaplan–Meier product restriction method, and the survival distribution of each group was compared by the log-rank test. The gray value of protein expression was detected by ImageJ software. Significance is indicated as follows: ^#^*P* > 0.05, *P < 0.05, **P < 0.01, ***P < 0.001.

## Results

### Low expression of SMARCC2 is associated with a poor prognosis of glioma and can inhibit the proliferation of glioblastoma cell lines. SMARCC2 can maintain the integrity of the SWI/SNF complex

**Fig. 1:**
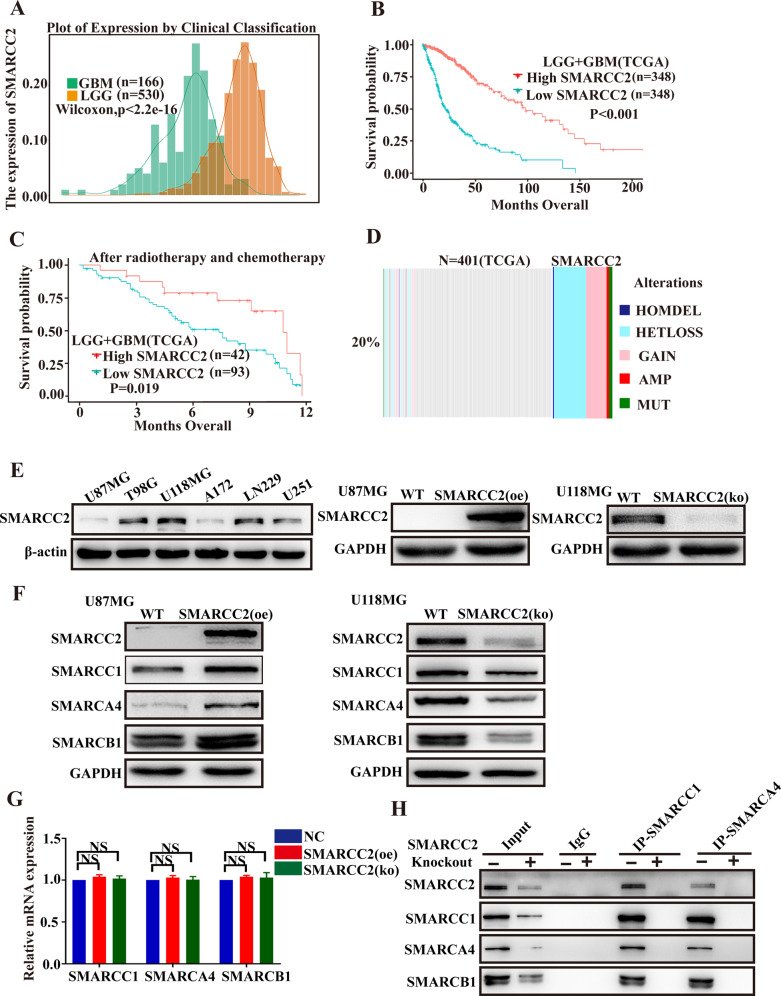
Low expression of SMARCC2 is associated with poor prognosis of glioma and can inhibit the proliferation of glioblastoma cell lines. SMARCC2 can maintain the integrity of the SWI/SNF complex. **A** Relative SMARCC2 expression in patient samples stratified according to the Verhaak classification based on TCGA database. **B**, **C** Overall survival analysis of patients with high (red) versus low (blue) SMARCC2 mRNA expression based on the TCGA database-GBM and TCGA database-LGG (**B**) also TCGA database-GBM + LGG after temozolomide radiotherapy and chemotherapy (**C**). **D** The mutation rate of SMARCC2 in GBM patients based on RNA-seq data from the TCGA database. **E** Western blotting of SMARCC2 indicated high expression in U118MG, T98G, and LN229, but low expression in U87MG, A172, and U251 cells (left). Oe-SMARCC2 adenovirus and CRISPR–cas-SMARCC2 sequences were used to upregulate and knockdown SMARCC2 protein levels (right). **F** Western blotting detected the changes of SMARCC1, SMARCA4 and SMARCB1 protein levels after up- or downregulation of SMARCC2 in U87MG and U118MG cells, respectively. **G** mRNA levels of SMARCC1, SMARCA4 and SMARCB1, were detected by qRT–PCR upon treatment with oeSMARCC2 in U87MG cells, or koSMARCC2 in U118MG cells. **H** Immunoprecipitation (IP) of the SWI/SNF complex subunit SMARCC2 from nuclear extracts of the koSMARFCC2 U118MG cell lines followed by immunoblotting for subunits SMARCB1, SMARCC1, SMARCA4. Data are expressed as mean ± SEM. ns not significant, *P < 0.05, **P < 0.01, ***P < 0.001.

SMARCC2, as the basic module of the SWI/SNF chromatin remodeling complex, stabilizes the whole complex. It has been widely studied in a variety of cancers [21], but its role in glioma is poorly understood. Therefore, this study analyzed the difference in SMARCC2 expression between low-grade glioma (LGG) and glioblastoma (GBM) in the TCGA database. We found that the expression of SMARCC2 was higher in LGG (Fig. 1A) and that the high expression of SMARCC2 tended to characterize a better prognosis of glioma (Fig. 1B). Strengthening the credibility of this conclusion, analysis of the survival statistics of 136 patients who underwent postoperative radiotherapy and chemotherapy also indicated that low expression of SMARCC2 was associated with poor prognosis of glioma (Fig. 1C). Next, data from 401 patients with gene mutation information were analyzed, and the mutation rate of SMARCC2 was found to be as high as 20% in gliomas, with loss of heterozygosity (HETLOSS) as a major component (Fig. 1D). To better understand SMARCC2, we performed Western blot analysis on six different glioblastoma (GBM) cell lines. U87MG cell lines with low expression of SMARCC2 were transfected with adenovirus (LV OE SMARCC2) overexpressing SMARCC2, and CRISPR–Cas specific knockout of SMARCC2 was performed in U118MG cell lines with high expression of SMARCC2 (Fig. 1E). Previous studies have shown that SMARCC2 constitutes the basic module of the SWI/SNF complex. We further studied glioblastoma cell lines and found that when SMARCC2 was specifically knocked out, western blot confirmed that the expression of other subunits of the complex (such as SMARCA4/SMARCC1/SMARCB1) was downregulated, and the re-expression of SMARCC2 resulted in a significant increase in the protein level of many SWI/SNF subunits (Fig. 1F). Interestingly, when SMARCC2 was knocked out, the mRNA content of other subunits (such as SMARCA4/SMARCC1/SMARCB1) did not change (1 G), indicating that the observed protein changes may be the result of posttranslational regulation. Further protein immunoprecipitation experiments (co-IP) showed that when SMARCC2 was specifically knocked out, the protein subunits no longer bound to one another, and the overall structure of the complex collapsed (Fig. 1H). To evaluate the tumor suppression function of SMARCC2, U87MG and U118MG cell lines were used with stable expression and knockout of SMARCC2 as models. Cell viability assays showed that U87MG cells overexpressing SMARCC2 had a significantly lower proliferation rate than those transfected with negative control virus carrying GFP (Supplementary Fig. 1SA). Similarly, compared with cells transduced with blank control plasmid (NC), cells transduced with SMARCC2 sgRNA plasmid (SMARCC2 (ko)) showed a higher proliferation rate (Supplementary Fig. 1SB). The plate cloning experiment and EdU experiment also confirmed that SMARCC2 could significantly inhibit the proliferation rate of the glioblastoma cell line (Supplementary Fig. 1S, C, D).

### SMARCC2 exerts a tumor-suppressive function in glioblastoma by changing the conformation of chromatin

**Fig. 2:**
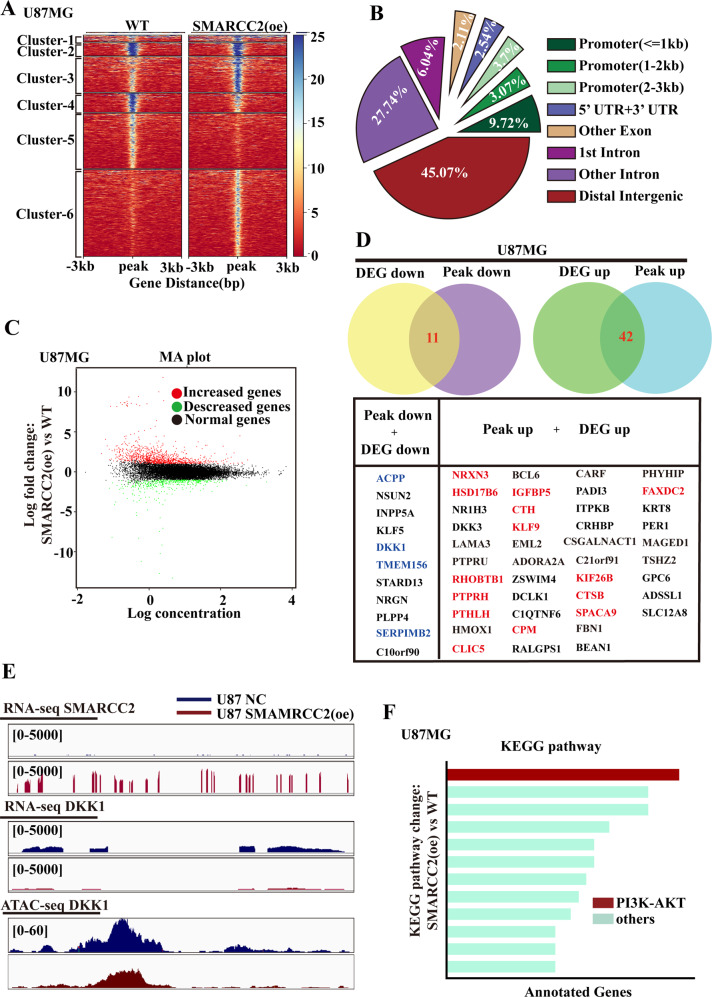
SWI/SNF alters cellular chromatin polarity. **A** ATAC-seq read-density heatmaps from U87MG cells overexpressing SMARCC2 for indicated durations (*n* = 2 biological replicates). **B** Genome-wide annotation of chromatin accessibility after overexpression of SMARCC2 in U87MG cells. **C** RNA-seq MA plot showing up (red)- and down (green)-regulated genes in the genome upon overexpression of SMARCC2 in U87MG cells. **D** Combined analysis of ATAC-seq and RNA-seq data to screen for genes with downregulated mRNA expression and reduced chromatin polarity (above, left), and another part of genes with upregulated mRNA expression and increased chromatin polarity (above, right). Specific gene names were presented in tabular form (under). **E** Representative screenshot in U87MG cells showing obviously changes in DKK1 upon SMARCC2 overexpression. **F** KEGG enrichment analysis of the above-changed genes.

The results above showed that SMARCC2 can control the proliferation rate of GBM cells, and the downregulation of SMARCC2 expression leads to an increase in cell proliferation. To determine the possible mechanism, transposase-accessible chromatin sequencing (ATAC-seq) analysis was performed on U87MG SMARCC2-overexpressing cells and WT control cells to evaluate whether SMARCC2 is needed to maintain chromatin accessibility. In this study, we observed 12,574 accessible regions in the genome, of which 2% (*n* = 2037) of chromatin regions were altered in SMARCC2-overexpressing cells (Fig. 2A). It is worth mentioning that more than 40% of the accessible region sites are located in distal regulatory regions, which are rich in enhancers (Fig. 2B). This result is consistent with a previous study showing that SWI/SNF complex enrichment plays a role in the distal enhancer region of genes [22]. Next, the transcriptomes of the SMARCC2 overexpression group and the control group were compared using RNA-seq. In U87MG cells, changes in expression were found in 1482 genes after overexpression of SMARCC2, of which 1041 were upregulated and 441 were downregulated (Fig. 2C). Comprehensive analysis of chromatin accessibility and gene expression datasets showed that the genes upregulated in SMARCC2-overexpressing cells were more related to the sites obtained by ATAC-seq, which means that SMARCC2 regulates the overexpression of tumor suppressor targets by promoting chromatin accessibility and then inhibits the transcription of oncogene targets. Next, ATAC-seq data and RNA-seq data were analyzed jointly. We observed 11 genes that were downregulated in the condensed chromatin regions and transcriptome, and 42 genes upregulated in the open chromatin regions and transcriptome (Fig. 2D). Among them, the transcripts that were downregulated or upregulated synchronously in U87MG cell are marked in blue and red, respectively, in the table. We then focused on the downregulated gene DKK1, which had the largest differential multiple. Visual characterization of the gene showed that the DKK1 gene was significantly downregulated after SMARCC2 overexpression (Fig. 2E). Pathway analysis of these SMARCC2 regulatory genes showed that the PI3K–Akt pathway, which is related to cell proliferation, was enriched (Fig. 2F).

### High expression of the target gene DKK1 is related to the poor prognosis of gliomas by promoting the proliferation of glioblastoma cells

**Fig. 3:**
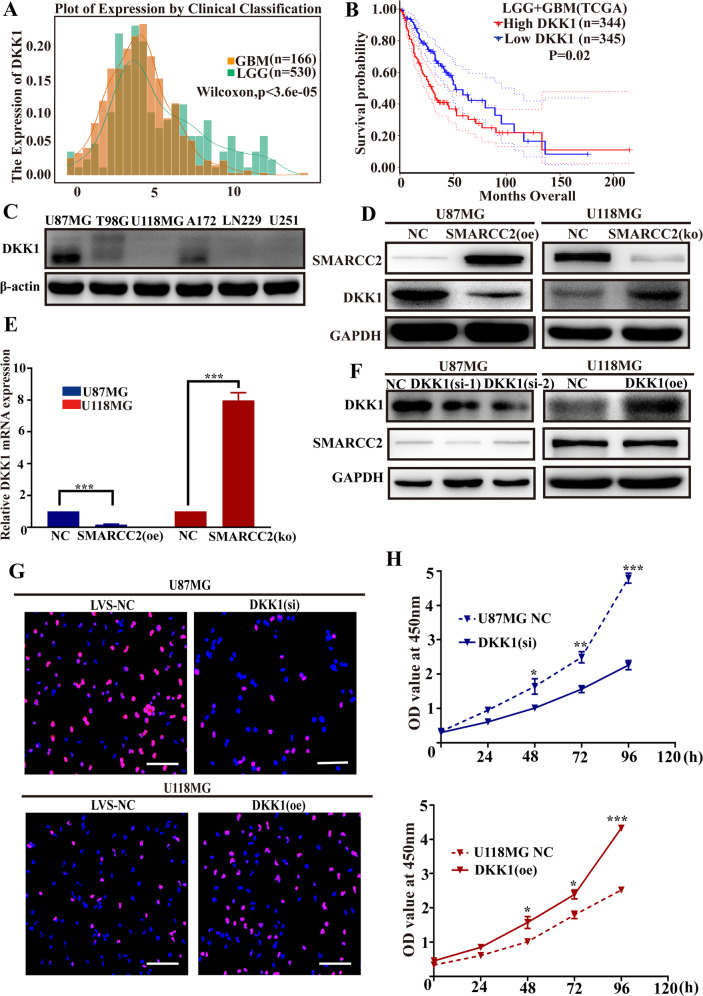
DKK1 is highly expressed in GBM and promotes the proliferation of glioblastoma cell lines. **A** Relative DKK1 expression in patient samples stratified according to the Verhaak classification based on the TCGA database. **B** Overall survival analysis of patients with high (red) versus low (blue) DKK1 mRNA expression based on the TCGA database-GBM and TCGA database-LGG. **C** Western blotting of DKK1 indicated high expression in U87MG, A172, but low expression in T98G, U118MG, LN229, and U251 cells. **D** The protein expression level of DKK1 was detected by western blot analysis in U87MG stably overexpressing SMARCC2 and U118MG cell line with a specific knockout of SMARCC2, respectively. **E** The mRNA expression level of DKK1 was detected by western blot analysis in U87MG, stably overexpressing SMARCC2 and U118MG cell line with a specific knockout of SMARCC2, respectively. **F** Changing the expression of DKK1 found that the protein expression of SMARCC2 did not change. **G** Changes in cell proliferation ability after overexpression and knockdown of DKK1 in U87MG cells and U118MG cells, respectively, detected by EDU staining. **H** Cell proliferation in siDKK1 U87MG cell or oeDKK1 U118MG cell were analyzed by CCK-8 assay at 24, 48, 72, 96, and 120 h after transfection. Data are expressed as mean ± SEM. ns not significant, *P < 0.05, **P < 0.01, ***P < 0.001.

The WNT signaling pathway inhibitor Dickkopf-1 (DKK1) is related to cancer progression [23]. However, its diagnostic and prognostic potential and value are rarely studied in glioblastoma. An increasing number of studies have shown that it can play an important role in cell proliferation through the PI3K–Akt pathway [24, 25]. In this study, the differential expression of DKK1 was evaluated in both low-grade gliomas (LGG *n* = 530) and high-grade gliomas (GBM *n* = 166) using The Cancer Genome Atlas (TCGA) dataset. The expression of DKK1 in GBM was significantly higher than that in LGG (Fig. 3A), and survival analysis showed that low expression of DKK1 was associated with a better prognosis in gliomas (Fig. 3B). Next, we characterized the negative correlation between the expression of DKK1 and SMARCC2 in different glioblastoma cell lines by western blotting (Fig. 3C). This analysis showed that the expression of DKK1 was significantly downregulated after overexpression of SMARCC2 in U87MG cells, while the expression of DKK1 was significantly upregulated in U118MG cells with specific SMARCC2 knockout (Fig. 3D). Further real-time quantitative PCR showed that the mRNA level of DKK1 was significantly negatively correlated with the expression of SMARCC2 (Fig. 3E), and changing the expression of DKK1 did not affect the protein expression of SMARCC2 (Fig. 3F). The above results show that SMARCC2 can inhibit the expression of the target gene DKK1 at the gene transcription level to exercise its tumor suppressor function in glioma. Next, EdU experiments were performed to observe the effects of knockdown and overexpression of DKK1 on cell proliferation in U87MG cells and U118MG cells. High expression of DKK1 significantly promoted the proliferation of glioblastoma cell lines (Fig. 3G); CCK-8 experiments also confirmed the same conclusion (Fig. 3H). This is consistent with a carcinogenic function for DKK1 in glioma.

### SMARCC2 inhibits the transcriptional function of the transcription factor EGR1 by partially closing the DKK1 promoter region

**Fig. 4:**
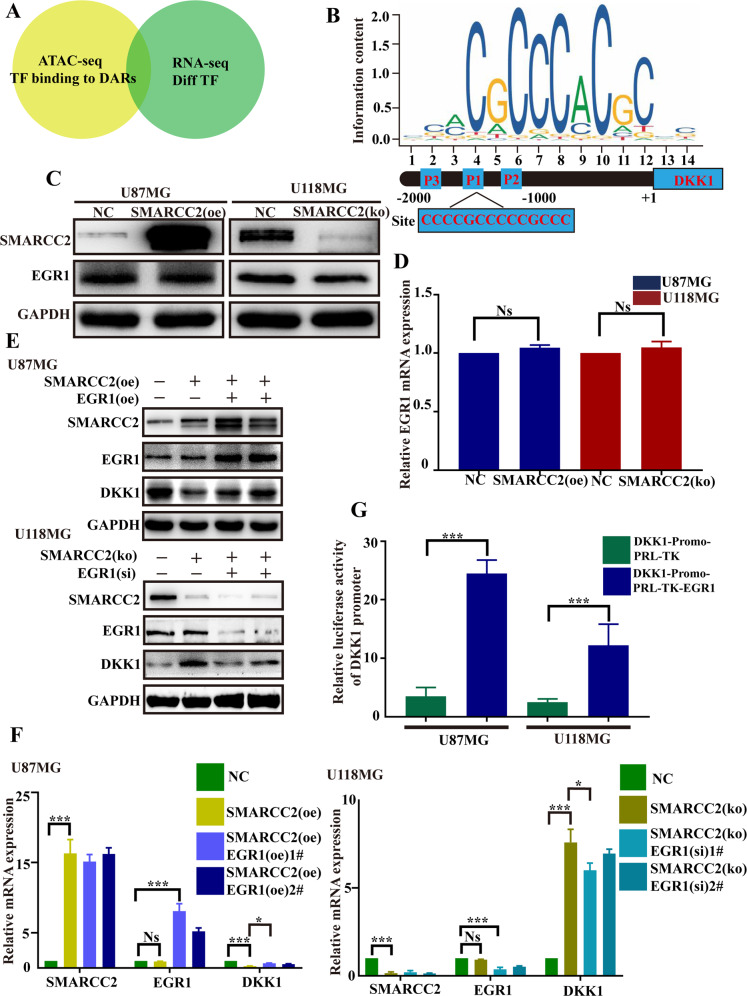
Bioinformatics screening of transcription factors potentially regulating the DKK1 promoter in glioma. **A** Combine ATAC-seq data and RNA-seq data to screen for possible transcription factors. **B** JASPAR motif logo of the predicted TF EGR1. **C** The protein level of EGR1 was unchanged after the change of SMARCC2 using a western blot. **D** The mRNA level of EGR1 was unchanged after the change of SMARCC2 using qRT–PCR. **E** Western blot was used to detect the protein levels of SMARCC2, DKK1, and EGR1 after Simultaneously alter the expression levels of SMARCC2 and EGR1 in glioma cells. Columns 3 and 4 show two independent replicates. **F** qRT–PCR was used to detect the mRNA levels of SMARCC2, DKK1, and EGR1 after change of SMARCC2 and EGR1 in glioma cells. 1# and 2# represent two independent replicate experiments. **G** Upregulation of EGR1promoted luciferase activity of luc-DKK1 in U87MG and U118MG cells. ***P < 0.001, compared with NC. Data are expressed as mean ± SEM. ns not significant, *P < 0.05, **P < 0.01, ***P < 0.001.

To explore the mechanism by which SMARCC2 negatively regulates the target gene DKK1, ATAC-seq and RNA-seq were applied to analyze the transcription factors that may bind to the DKK1 promoter (Fig. 4A). The results showed that the transcription factor EGR1 is most likely to bind to the DKK1 promoter sequence “CCCCGCCCCCCGCCC” (Fig. 4B). Interestingly, the change in SMARCC2 expression did not affect the expression of the EGR1 transcription factor at the protein or mRNA level (Fig. 4C, D). This result shows that SMARCC2 does not affect the transcriptional expression of the target gene DKK1 by affecting the expression of the transcription factor EGR1. With reference to the ATAC-seq data, we speculated that SMARCC2 closes the promoter region of DKK1 by chromatin remodeling, thus inhibiting the binding of the transcription factor EGR1 and then inhibiting the transcription of DKK1. To test this hypothesis, we overexpressed EGR1 and knocked down EGR1 in the U87MG cell line, stably overexpressing SMARCC2 and the U118MG cell line with specific knock out of SMARCC2 to observe the expression changes in the target gene DKK1. Western blot and Q-PCR experiments showed that when SMARCC2 negatively regulated the changes in the target gene DKK1 at both the protein and mRNA levels, the addition of EGR1 reversed this impact to varying degrees; interestingly, the effect was not completely reversed (Fig. 4E, F). On the one hand, these results indicated that EGR1 can affect the expression of DKK1 at the transcriptional level. However, SMARCC2 only partially closes the DKK1 promoter region. Therefore, when SMARCC2 is overexpressed, the expression of DKK1 is not completely knocked out. After overexpression of EGR1 in SMARCC2-overexpressing cell lines, the expression of DKK1 was partially reversed. A subsequent double luciferase reporter experiment showed that the fluorescence intensity was significantly enhanced when the transcription factor EGR1 was overexpressed (Fig. 4G), indicating that EGR1, as a transcription factor in the DKK1 promoter region, plays an important role in the transcription process of DKK1.

### The functional integrity of SMARCC2 depends most on its SWIRM domain

**Fig. 5:**
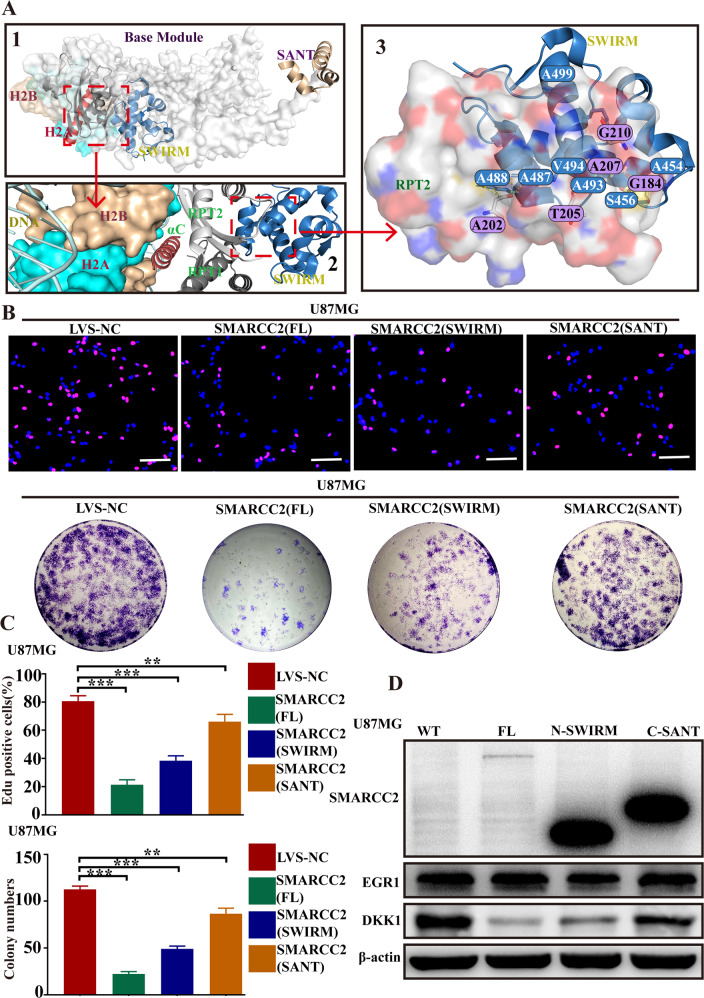
The functional integrity of SMARCC2 may depend more on the SWIRM domain. **A** shows the binding model of SWI/SNF and nucleosome (1), and zoom in to show that the SWIRM domain of SMARCC2 binds to the nucleosome via the RPT2 andαC domain of SMARCB1 (2). Further docking analysis revealed the amino acid site where the SWIRM domain and the RPT2 domain bind (3). **B** Plasmids containing the full-length domain (AA 1–1214), N-SWIRM domains (1–580 AA), and C-SANT domain (530–1214 AA) of SMARCC2 were transfected into U87MG cells.Cells were subsequently tested for proliferative capacity using EDU and colony-forming assay. **C**, **D** The mRNA and protein expression levels of SMARCC2, EGR1 and DKK1 were detected by qRT–PCR(C) and western blot (**D**) in U87MG cells transfected with the above plasmids, respectively. Data are expressed as mean ± SEM. ns not significant, *P < 0.05, **P < 0.01, ***P < 0.001.

Previous studies have shown that SMARCC2, as the basic subunit of the chromatin remodeling complex SWI/SNF, contains two functional domains: SWIRM (Ile424–Thr521) and SANT (Ser596–Pro647) [26, 27]. First, the positions of the two SMARCC2 domains, SWIRM and SANT, were analyzed in the whole SWI/SNF complex through Python. The SWIRM domain is closer to the nucleosome, while the SANT domain is located at the distal end (Fig. 5A1). Therefore, we focused on the SWIRM domain, and further structural analysis showed that two RPT domains and one C-terminal portion of the SWIRM domain and SMARCB1 subunit α helix (αC) bind to histone octamers together. Using PyMOL software to analyze its mode of action, we found that amino acid residues S456, A484, A488, A493, V494, and A499 of SMARCC2 formed hydrogen bond interactions with amino acid residues A202, T205, A207, G184, and G210 of SMARCB1, respectively (Fig. 5A2–3); this special structural relationship also reflects that the SWIRM domain may play a more critical role in chromatin remodeling than the SANT domain. To test this hypothesis, we transfected full-length plasmids with full-length SMARCC2 (SMARCC2-FL), the SWIRM domain (SMARCC2-SWIRM) and the SANT domain (SMARCC2-SANT) into U87MG cells with low SMARCC2 expression and then observed the cell proliferation phenotype. EdU and plate cloning experiments showed that the cells exhibited significant proliferation inhibition after transfection with the full-length plasmid. Although the cells transfected with the SWIRM domain did not reach the same level of cell proliferation inhibition as the full-length plasmid group, its effect was significantly better than that of the cells transfected with the SANT domain (Fig. 5B). We conducted three groups of independent repeated experiments to statistically analyze the inhibition of specific SMARCC2 full-length protein, SWIRM domain and SANT domain on cell proliferation and obtained the same results (Fig. 5C). Furthermore, we detected the effects of three groups of plasmids against SMARCC2 and a group of negative control plasmids on the protein expression levels of the downstream target gene DKK1 and transcription factor EGR1. The results showed that the downregulation of DKK1 expression by the SWIRM domain was significantly better than that by the SANT domain, and the inhibition of DKK1 by the SMARCC2 full-length protein was the most significant (Fig. 5D). The above results suggest that the functional integrity of SMARCC2 may depend more on the SWIRM domain, which is closer to the nucleosome in space, which provides a prospect for the design of targeted drugs for the treatment of glioblastoma in the future.

### SMARCC2 can significantly reduce tumor proliferation in nude mice by inhibiting the PI3K–Akt pathway

**Fig. 6:**
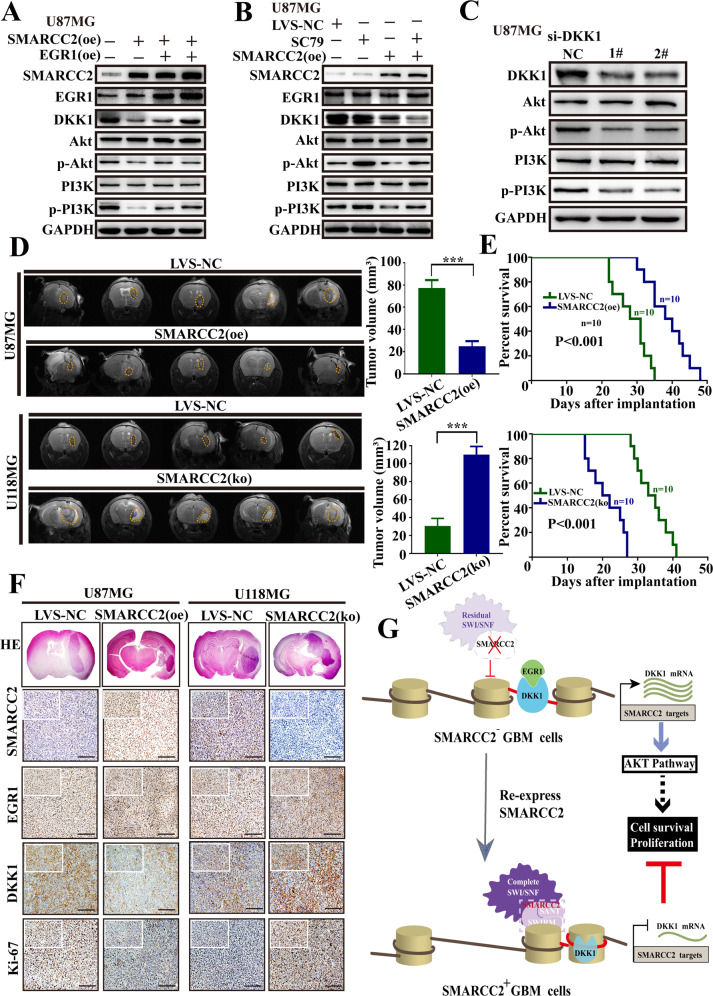
SMARCC2 increases tumor invasion upon intracranial tumor transplantation in nude mice. **A** EGR1 was overexpressed in U87MG cells that stably overexpressed SMARCC2, and the expression levels of PI3K–AKT pathway-related proteins were detected by western blot. **B** Using PI3K–AKT pathway inhibitor SC79 in U87MG cells stably overexpressing SMARCC2, the expression levels of PI3K–AKT pathway-related proteins were detected by western blot. **C** Inhibition of the PI3K–AKT pathway was observed using Western blot assay after DKK1 knockdown in U87MG cells. **D** Coronal section of MRI scan was used to evaluate the tumor growth after intracranial tumor transplantation in nude mice. The tumor volume (mean ± SD) was statistically analyzed as a histogram. **E** The follow-up data of the nude mice with en suite tumorigenesis were recorded to process the survival analysis. **F** IHC detection was used to evaluate the expression of SMARCC2, EGR1, DKK1, and Ki-67 in intracranial tumors of different groups. Scale bar, 100 mm. **G** Schematic drawing indicating the mechanism by which SMARCC2 stabilizes the SWI/SNF complex to regulate chromatin structure to downregulate DKK1 and suppress glioblastoma proliferation through the PI3K–AKT pathway. Data are expressed as mean ± SEM. ns not significant, *P < 0.05, **P < 0.01, ***P < 0.001.

Pathway enrichment analysis of the differentially expressed genes identified by ATAC-seq and RNA-seq showed that the PI3K–AKT pathway was significantly enriched. Therefore, we further evaluated the potential role of the PI3K–Akt signaling pathway in GBM cells in SMARCC2-induced cell proliferation. The results showed that SMARCC2 significantly reduced the ratio of p-PI3K/PI3K and p-Akt/Akt, indicating the inhibition of the PI3K–Akt signaling pathway (Fig. 6A). Interestingly, the activation of the PI3K–Akt signaling pathway by PI3K–Akt pathway promoter sc79 was eliminated by SMARCC2 overexpression, which means that there is a link between SMARCC2 and PI3K–Akt inhibition (Fig. 6B). In addition, the overexpression of DKK1 significantly activated the PI3K–Akt signaling pathway (Fig. 6C). Therefore, we speculate that SMARCC2 acts as an upstream signaling factor by affecting the expression of downstream target genes to inhibit the activation of the PI3K–Akt signaling pathway but not EGR1. The promotion of the PI3K–Akt pathway after specific knockdown of SMARCC2 was validated in the U118MG cell line (Supplementary Fig. S2). The U87MG cell line SMARCC2 (oe) with stable overexpression of SMARCC2 and the U118MG cell line SMARCC2 (ko) with a specific knockout of SMARCC2 were selected for intracranial tumorigenesis experiments in nude mice. MRI of SMARCC2 (oe) mice showed significantly smaller tumors after implantation. In contrast, downregulation of SMARCC2 significantly increased tumor volume (Fig. 6D). Survival analysis showed that low SMARCC2 expression was closely related to poor prognosis in transplanted nude mice (Fig. 6E). In addition, immunohistochemical detection showed that the expression levels of SMARCC2 and DKK1 were in opposition, while the expression of EGR1 was relatively stable. Ki-67 staining also showed that specific knockout of SMARCC increased cell proliferation (Fig. 6F).

## Discussion

Here, we report that the SMARCC2 subunit of the SWI/SNF chromatin remodeling complex can significantly inhibit the proliferative ability of glioblastoma (GBM) cell lines. Our mechanistic studies show that SMARCC2 represses the expression of the oncogene DKK1 by stabilizing the SWI/SNF complex at the promoters of genes throughout the genome of GBM cell lines, which in turn inhibits cell proliferation through the PI3K–AKT pathway, suggesting that SMARCC2 is a potential target for the treatment of glioblastoma.

The mammalian SWI/SNF complex is a combinatorial assembly consisting of at least 13 subunits encoded by 29 genes [28]. Therefore, we first verified whether SMARCC2 affects the overall assembly of the SWI/SNF complex in GBM cell lines. First, our evidence shows that SMARCC2, as the basic subunit of the complex, can stabilize the function of the complex. Western blotting proved that when SMARCC2 was specifically knocked out, the expression levels of residual subunits, such as SMARCA4/SMARCB1/SMARCC1, were downregulated. Overexpression of SMARCC2 enhanced the stability and function of SWI/SNF. Interestingly, however, its mRNA levels did not change significantly, suggesting that the observed changes are the result of posttranslational regulation. Further protein coimmunoprecipitation experiments showed that when SMARCC2 was deleted, the residual subunits were not sufficiently stable to form complexes and disintegrated.

In some forms of cancer, SWI/SNF is associated with tumor progression, while in others, SWI/SNF is associated with tumor suppression [9, 29–31]. Different subunits of the complex also appear to have different effects in different cancers [32]. With mutations in SWI/SNF gene being found in nearly 25% of cancers, the question naturally arises whether such mutations have prognostic significance. These mutations have indeed been linked to a worse prognosis across several cancer types. This study found that higher-grade gliomas had lower SMARCC2 expression and that higher expression was significantly associated with better prognosis. Subsequent cell function experiments also demonstrated the tumor suppressor function of SMARCC2 subunits in GBM cell lines.

Second, a key observation in our study is the convincing evidence that SMARCC2 is associated with distal intergenic regions in GBM cell lines overexpressing SMARCC2. Combined with previous findings [33–36]. The functional implications of the hyper-diversity of SWI/SNF subunit composition is not entirely clear, but the different subfamilies have distinct location profiles across enhancers, promoters and gene bodies, and their distinctive compositions are thought to provide specificity in interactions with transcription factors and other chromatin regulators. This observation leads us to speculate that in glioma, the complete SWI/SNF complex associates with the distal enhancer region of the gene to regulate gene transcription. On the other hand, after upregulating the expression of SMARCC2, we observed that the gene promoter region of the downstream target gene DKK1 was significantly turned off so that the binding of the transcription factor EGR1 was inhibited, although this inhibition was not complete. Even when SMARCC2 was overexpressed in the highly significant U87MG cell line, DKK1 expression was still observed. This finding provides us with new insight that SMARCC2 may exert a tumor suppressor function not through a single downstream target gene but through the corepression of multiple target genes, such as ACPP/TMEM156/KLF5.

Functional experimental analysis of the two conserved functional domains of SMARCC2, SWIRM, and SANT, showed that the function of the SWIRM domain is closer to that of the complete SMARCC2, which we speculate is large because the SWIRM domain is closer to the bottom of the nucleosome and thus can bind to the nucleosome through the αC subunit of SMARCB1 and retain its chromatin remodeling function. This also reveals the conservation of the SWI/SNF complex during development and evolution from another perspective [37–39], showing that only a single functional domain must be retained for tumor suppressor activity in GBM.

Loss of SWI/SNF complex components has been reported to promote the malignant progression of rhabdomyosarcoma and ovarian cancer by activating the PI3K–AKT pathway [40, 41]. Dickkopf-1 (DKK1) was originally identified as an antagonist of Wnt signaling [42] and can also bind to cytoskeleton-associated protein 4 (CKAP4) [25], which was originally identified as an endoplasmic reticulum (ER) protein [43]. The DKK1-CKAP4 pathway is activated in several human cancers and promotes cell proliferation by activating signaling through the kinases PI3K and AKT. We observed in our study that the PI3K–AKT pathway was significantly inhibited when SMARCC2 was overexpressed; however, the PI3K–AKT promoter SC79 only partially reversed these tumor suppressor effects, suggesting that other epigenetic factors may be involved. Taken together, these observations suggest that SMARCC2 may act as a tumor suppressor in GBM along with other epigenetic factors. This possibility requires further investigation.

One type of vulnerability has been clearly recognized based on findings that mutations in certain genes encoding SWI/SNF subunits often create specific dependencies on genes encoding other SWI/SNF subunits. Such data suggest a model whereby subunit mutations do not fully inactivate SWI/SNF function but rather result in aberrant cell function owing to a reliance on the activity of alternative residual SWI/SNF complexes. Several molecules capable of inhibiting SWI/SNF ATPase activity have been identified. For example, an orally available allosteric inhibitor of both SMARCA2 and SMARCA4 has been discovered and has demonstrated antiproliferative activity in a mouse xenograft model of SMARCA4-mutant lung cancer [44]. Importantly, the development of therapeutic strategies to target aberrant residual SWI/SNF complex warrants consideration since some SMARCA4-mutant cancers, such as SCCOHTs and a subset of non-small-cell lung carcinomas, lack expression of SMARCA2 and can, therefore, grow in the absence of both ATPase subunits [45]. In the present study, we demonstrate that the residual SWI/SNF group after SMARCC2 is specifically knocked out leads to the disorder of the genome, and perhaps the development of degraders targeting the residual SWI/SNF complex may be beneficial to SMARCC2-deficient glioblastoma.

Given the findings that SWI/SNF complexes contribute to the regulation of enhancer function and, as part of this role, facilitate the acetylation of H3K27, investigation of compounds that alter histone acetylation levels are obviously of interest in the context of SWI/SNF-aberrant cancers [46]. Loss-of-function mutations in ARID1A often co-occur with activating mutations in PI3K, AKT or mTOR or with loss of PTEN, which all result in upregulation of PI3K/AKT signaling, However, the finding that the small-molecule SMARCA4/2 inhibitor PFI-3 has a promising therapeutic activity in preclinical models of PTEN-deficient prostate cancer suggests additional complexity in the relationship between the PI3K/AKT axis and SWI/SNF function [47]. Our study showed that SMARCC2 could significantly inhibit the PI3K/AKT pathway in glioblastoma cell lines by targeting DKK1, therefore, targeting DKK1 degraders and PI3K/AKT pathway inhibitors may be a new idea for the clinical treatment of glioblastoma.

To our knowledge, this is the first study to demonstrate a role for SMARCC2 in glioblastoma at the level of physical chromatin accessibility via the SWI/SNF complex. Our findings not only suggest that SMARCC2 may serve as a prognostic indicator in glioblastoma patients but also raise the possibility of the clinical benefit of PI3K–AKT inhibition in patients with SMARCC2-low or null cancers.

## Supplementary information

supplementary material file

Original Data File

Reproducibility Checklist

## Data Availability

All data generated or analyzed during this study are included in this published article and its Supplementary files and are available from the corresponding authors on request.
