# Correction: SMARCC2 mediates the regulation of DKK1 by the transcription factor EGR1 through chromatin remodeling to reduce the proliferative capacity of glioblastoma

**DOI:** 10.1038/s41419-022-05498-x

**Published:** 2022-12-12

**Authors:** Chiyang Li, Tong Wang, Junwei Gu, Songtao Qi, Junjie Li, Lei Chen, Hang Wu, Linyong Shi, Chong Song, Hong Li, Liwen Zhu, Yuntao Lu, Qiang Zhou

**Affiliations:** 1 Department of Neurosurgery, Southern Medical University, Guangzhou, China; 2 Nanfang Neurology Research Institution, Nanfang Hospital, Southern Medical University, Guangzhou, China; 3 Nanfang Glioma Center, Guangzhou, China; 4 Department of Hematology, Nanfang Hospital, Southern Medical University, 510000 Guangzhou, Guangdong P.R. China

**Keywords:** CNS cancer, Chromosomes

Correction to: *Cell Death and Disease* 10.1038/s41419-022-05439-8, published online 23 November 2022

The original version of this article contained a mistake. Professor Yuntao Lu contributed a lot for this study, and he also funded the progress of the article, and provided the ideas for the article. Therefore, the authors would like to add the note “Professor Qiang Zhou and Professor Yuntao Lu as co-corresponding authors”, and note Yuntao Lu as the co-corresponding author of this article (Email: lllu2000yun@gmail.com). The original article has been corrected.

