# Correction: SMARCC2 mediates the regulation of DKK1 by the transcription factor EGR1 through chromatin remodeling to reduce the proliferative capacity of glioblastoma

**DOI:** 10.1038/s41419-023-05920-y

**Published:** 2023-07-11

**Authors:** Chiyang Li, Tong Wang, Junwei Gu, Songtao Qi, Junjie Li, Lei Chen, Hang Wu, Linyong Shi, Chong Song, Hong Li, Liwen Zhu, Yuntao Lu, Qiang Zhou

**Affiliations:** 1 Department of Neurosurgery, Nanfang Hospital, Southern Medical University, Guangzhou, China; 2 Nanfang Neurology Research Institution, Nanfang Hospital, Southern Medical University, Guangzhou, China; 3 Nanfang Glioma Center, Guangzhou, China; 4 Department of Hematology, Nanfang Hospital, Southern Medical University, 510000 Guangzhou, Guangdong China

**Keywords:** CNS cancer, Chromosomes

Correction to: *Cell Death and Disease* 10.1038/s41419-022-05439-8, published online 23 November 2022

In this article the affiliation of Qian Zhou has not given correctly. It should read: Department of neurosurgery, Nanfang Hospital, Southern Medical University, Guangzhou, China

The original article has been corrected.

