# Supplementary material for: SMARCC2 mediates the regulation of DKK1 by the transcription factor EGR1 through chromatin remodeling to reduce the proliferative capacity of glioblastoma
Source: Cell Death Dis. 2022 Nov 23;13(11):990. doi: 10.1038/s41419-022-05439-8 (PMC9684443; doi:10.1038/s41419-022-05439-8)
Supplement: Supplementary file 1 — supplementary material file [file 41419_2022_5439_MOESM1_ESM.docx]

**Figure 1S**

**Figure 1S** SMARCC2 inhibits GBM cell line proliferation.**A and B** Cell proliferation in oeSMARCC2(A) U87MG cell(A) or koSMARCC2(B) U118MG cell were analyzed by CCK-8 assay at 24, 48, 72,96, and 120 h after transfection. **C and D** colony forming assay:SMARCC2 overexpression via Adenovirus transfection impairs cell proliferation(above) and SMARCC2 deletion via CRISPR-Cas9 increases cell proliferation(under). Figures are representative of three independent experiments. Data are expressed as mean ± SEM. ns not significant, *p < 0.05, **p < 0.01, ***p < 0.001(D). **E and F** Changes in cell proliferation ability after overexpression and knockdown of SMARCC2 in U87MG cells and U118MG cells, respectively, detected by EDU staining.Data are expressed as mean ± SEM. ns not significant, *p < 0.05, **p < 0.01, ***p < 0.001

**Figure 2S**

**Figure 2S** SMARCC2 inhibits the PI3K-AKT pathway by downregulating DKK1.**A** EGR1 was knocked down in U118MG cells stably knocked out SMARCC2, and the expression levels of PI3K-AKT pathway-related proteins were detected by Western blot. **B** Using the PI3K-AKT pathway promoter MK2206 in U118MG cells stably knocked out SMARCC2, the expression levels of PI3K-AKT pathway-related proteins were detected by Western blot. **C** Promotion of the PI3K-AKT pathway was observed using western blot analysis following DKK1 overexpression in U118MG cells.
