# Supplementary figures and images for: SMARCC2 mediates the regulation of DKK1 by the transcription factor EGR1 through chromatin remodeling to reduce the proliferative capacity of glioblastoma
Source: Cell Death Dis. 2022 Nov 23;13(11):990. doi: 10.1038/s41419-022-05439-8 (PMC9684443; doi:10.1038/s41419-022-05439-8)

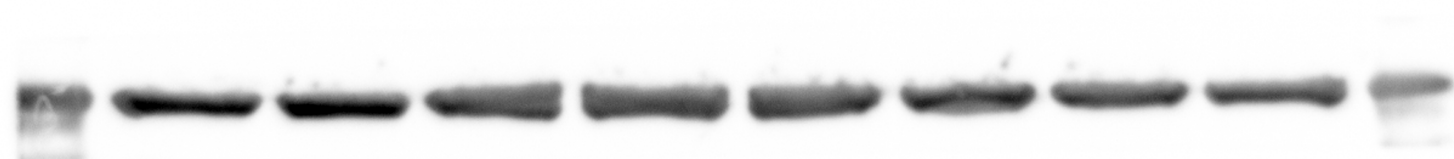

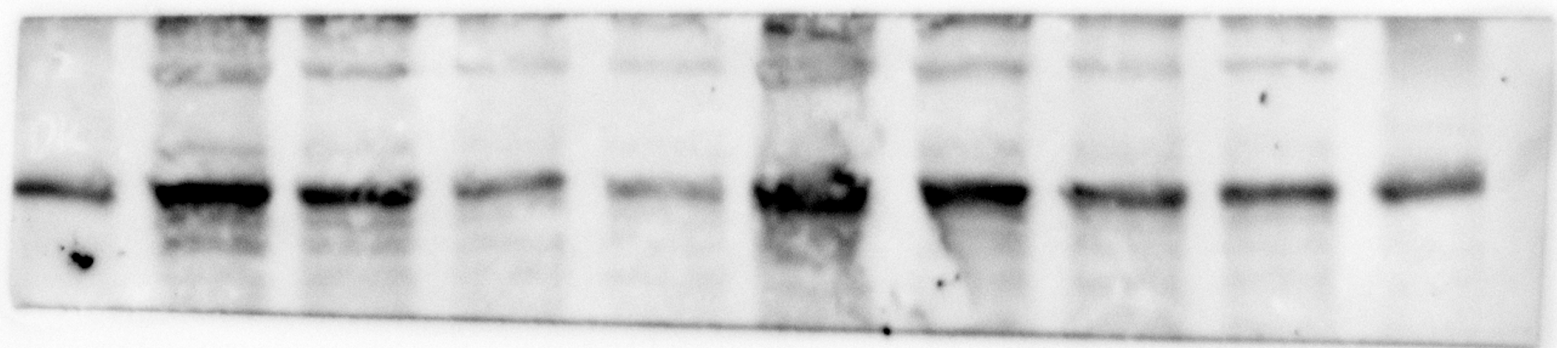

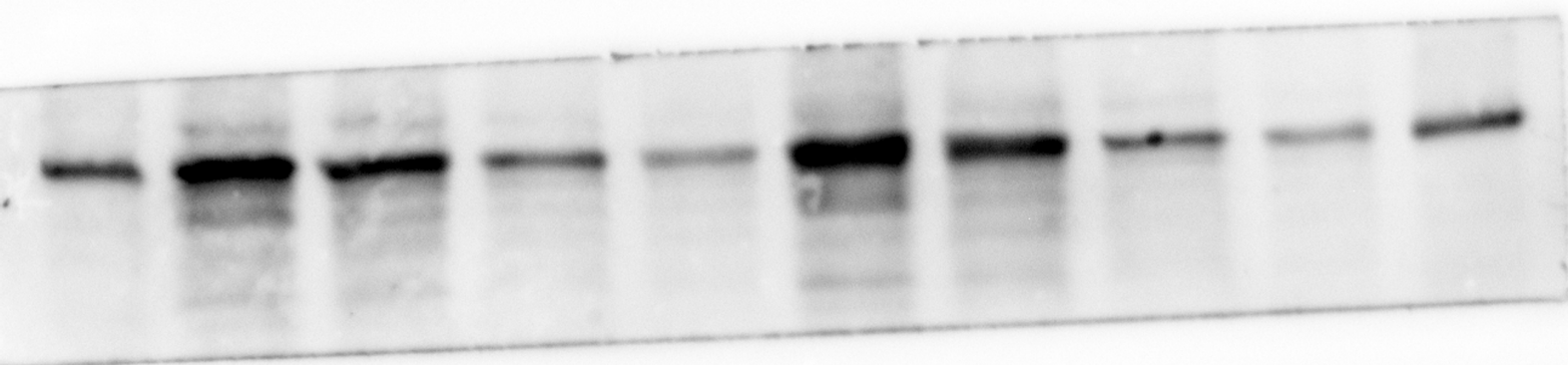

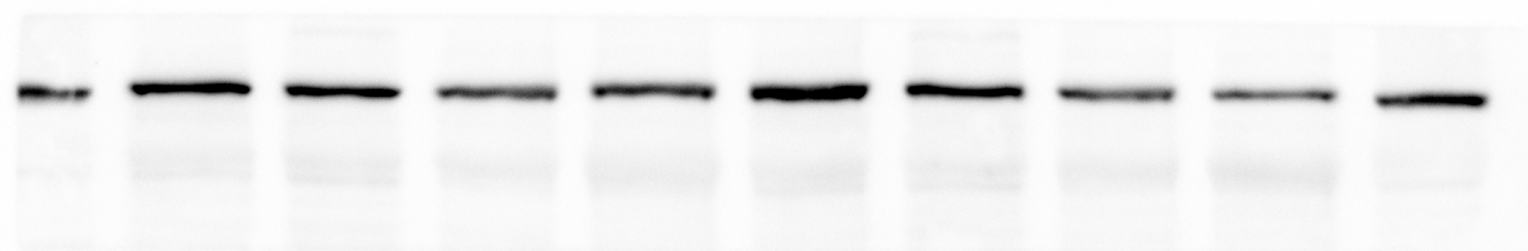

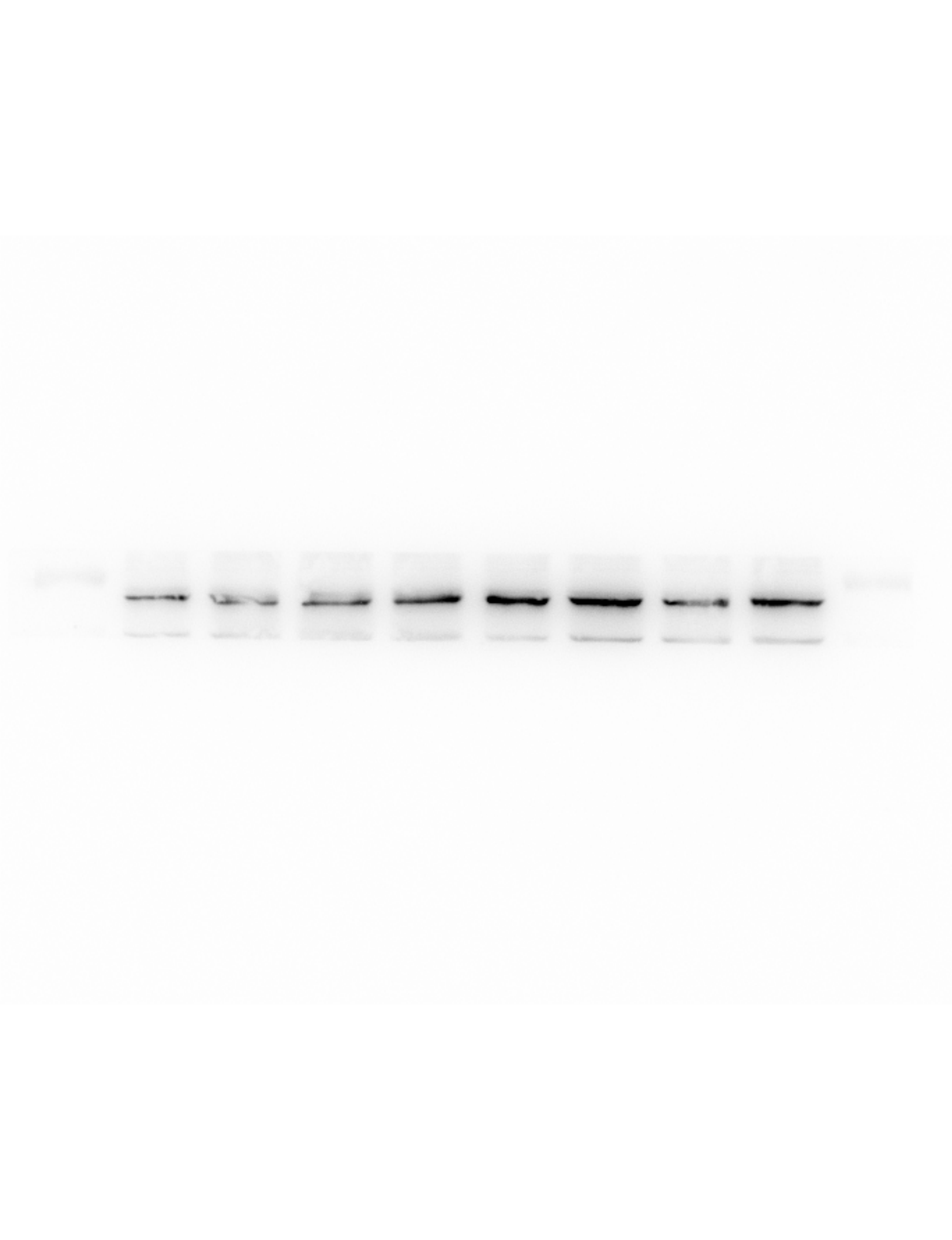

— — — — —

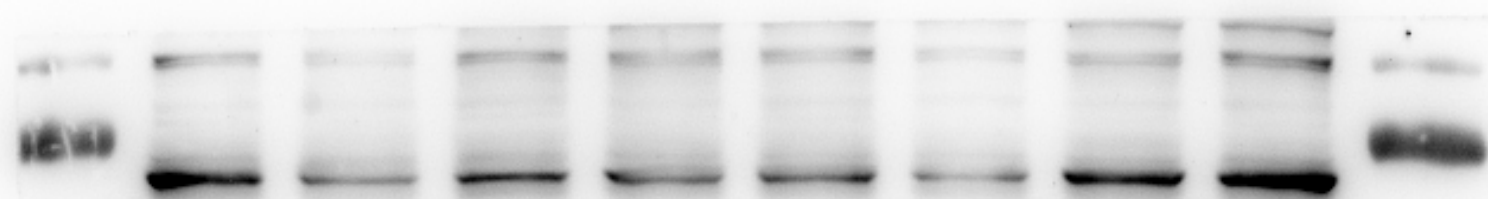

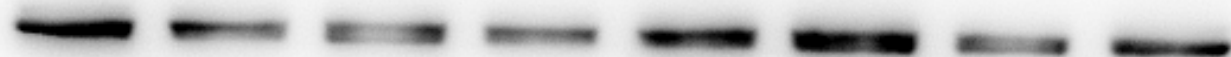

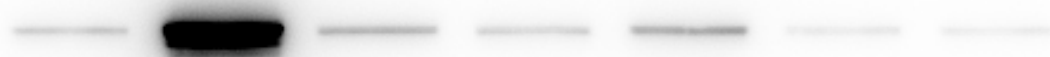

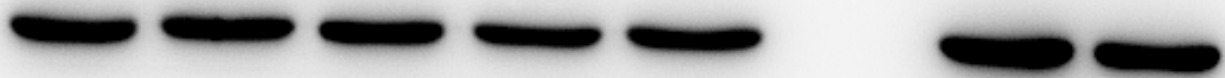

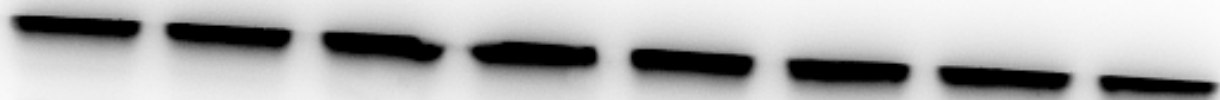

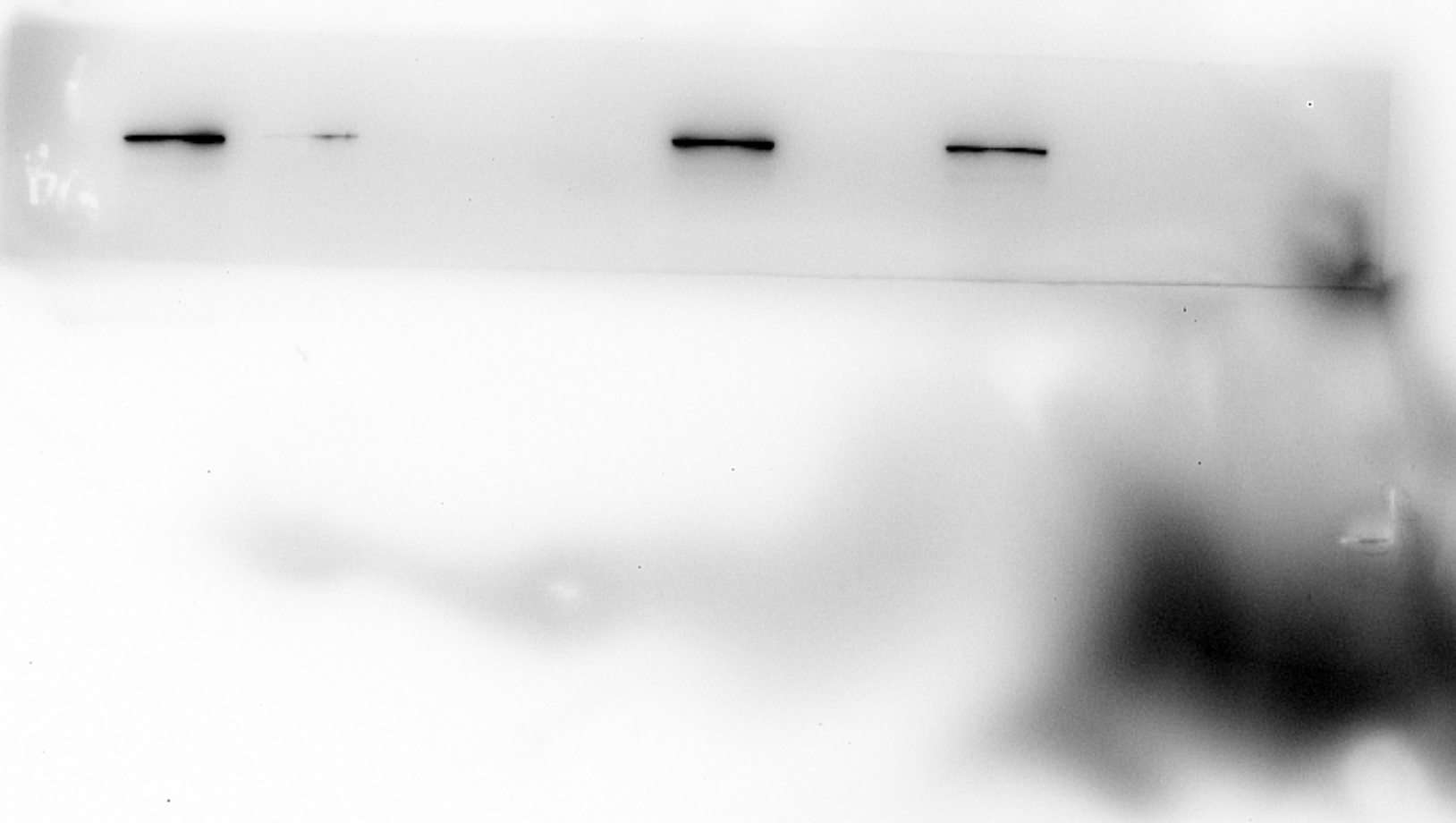

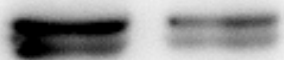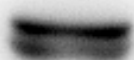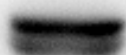

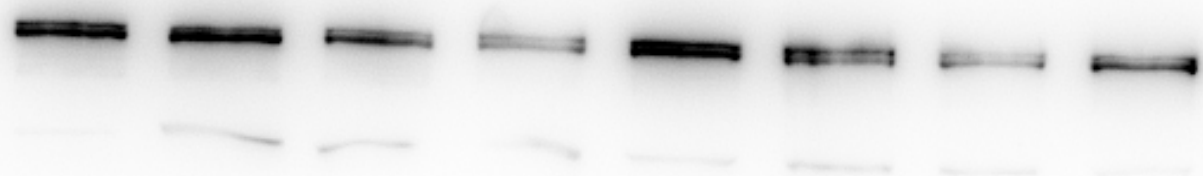

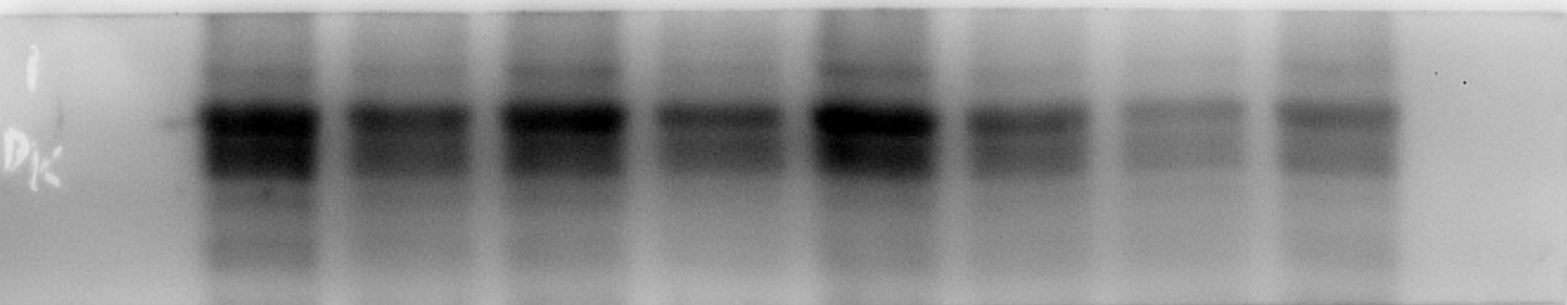

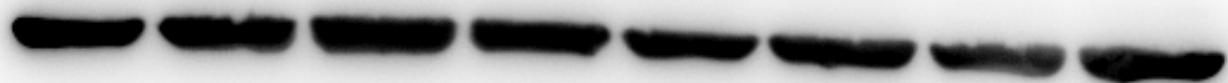

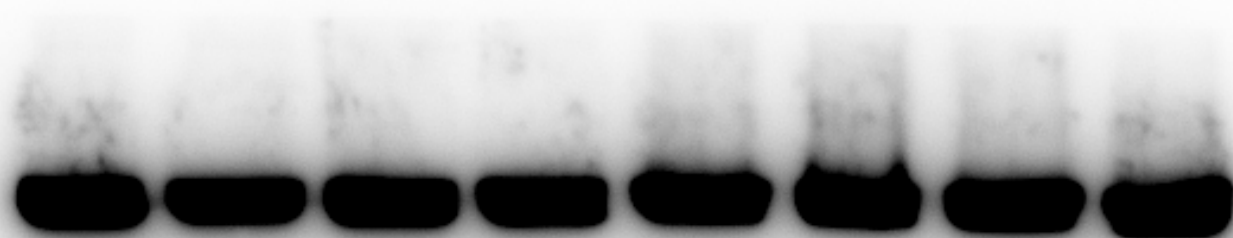

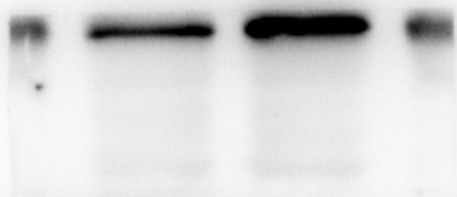

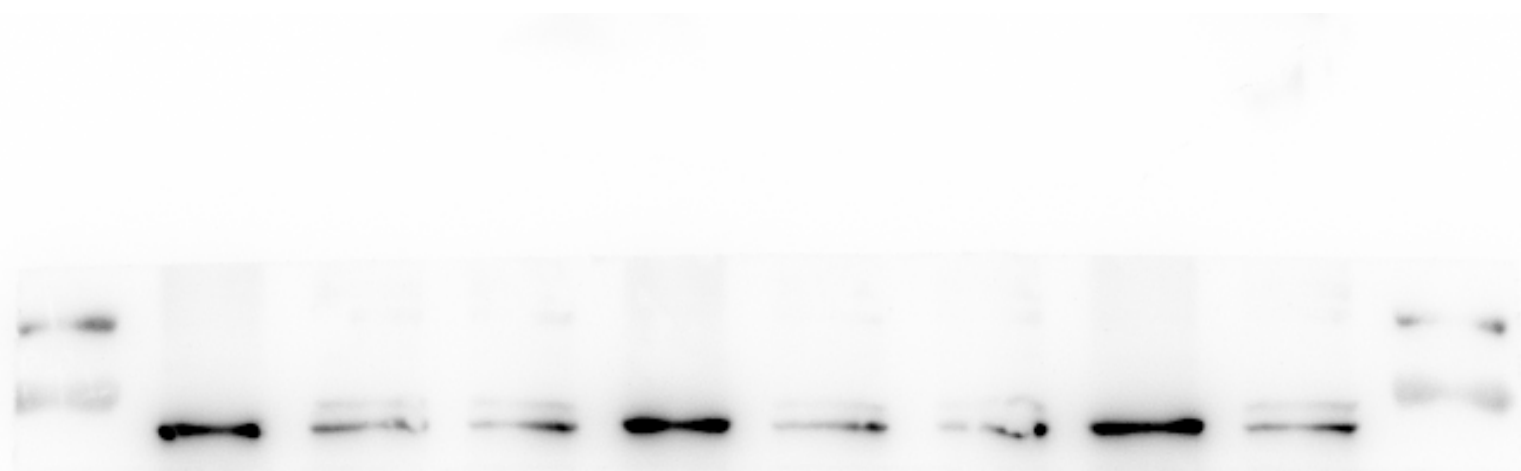

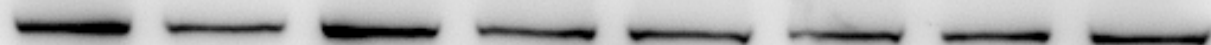

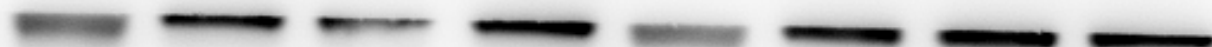

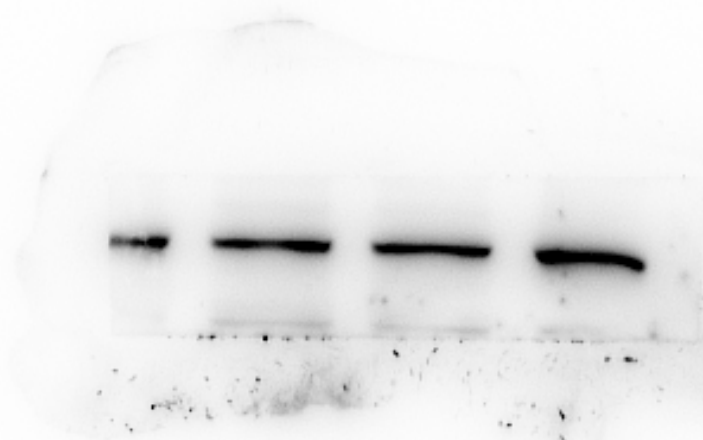

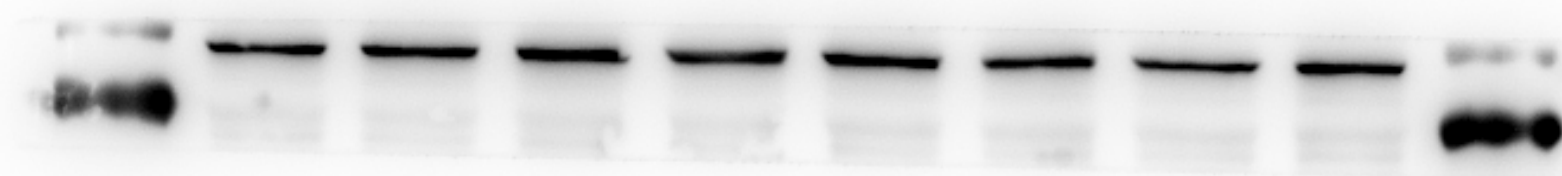

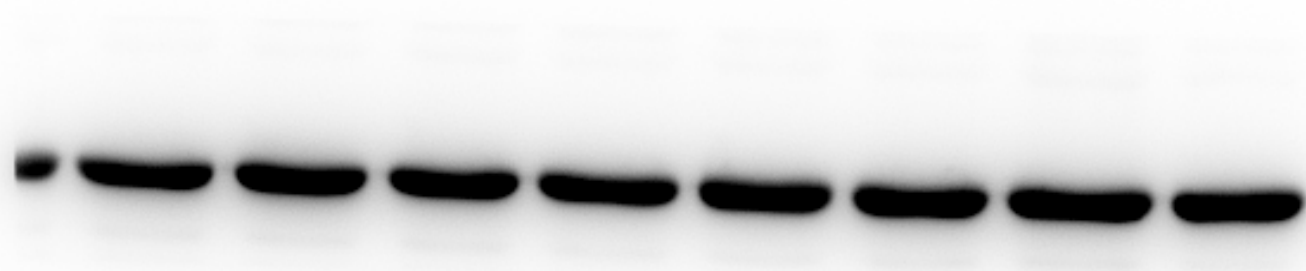

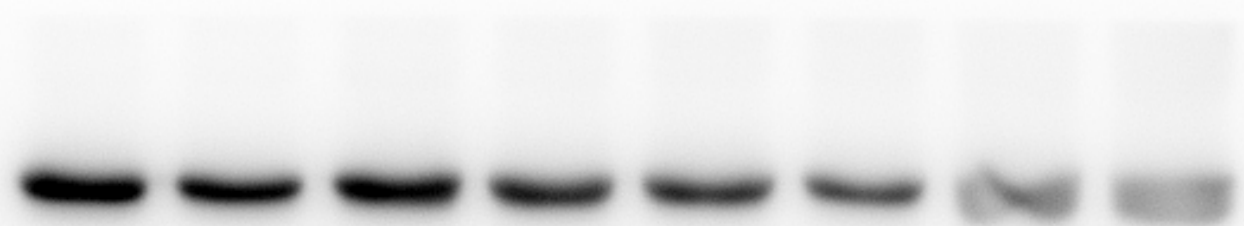

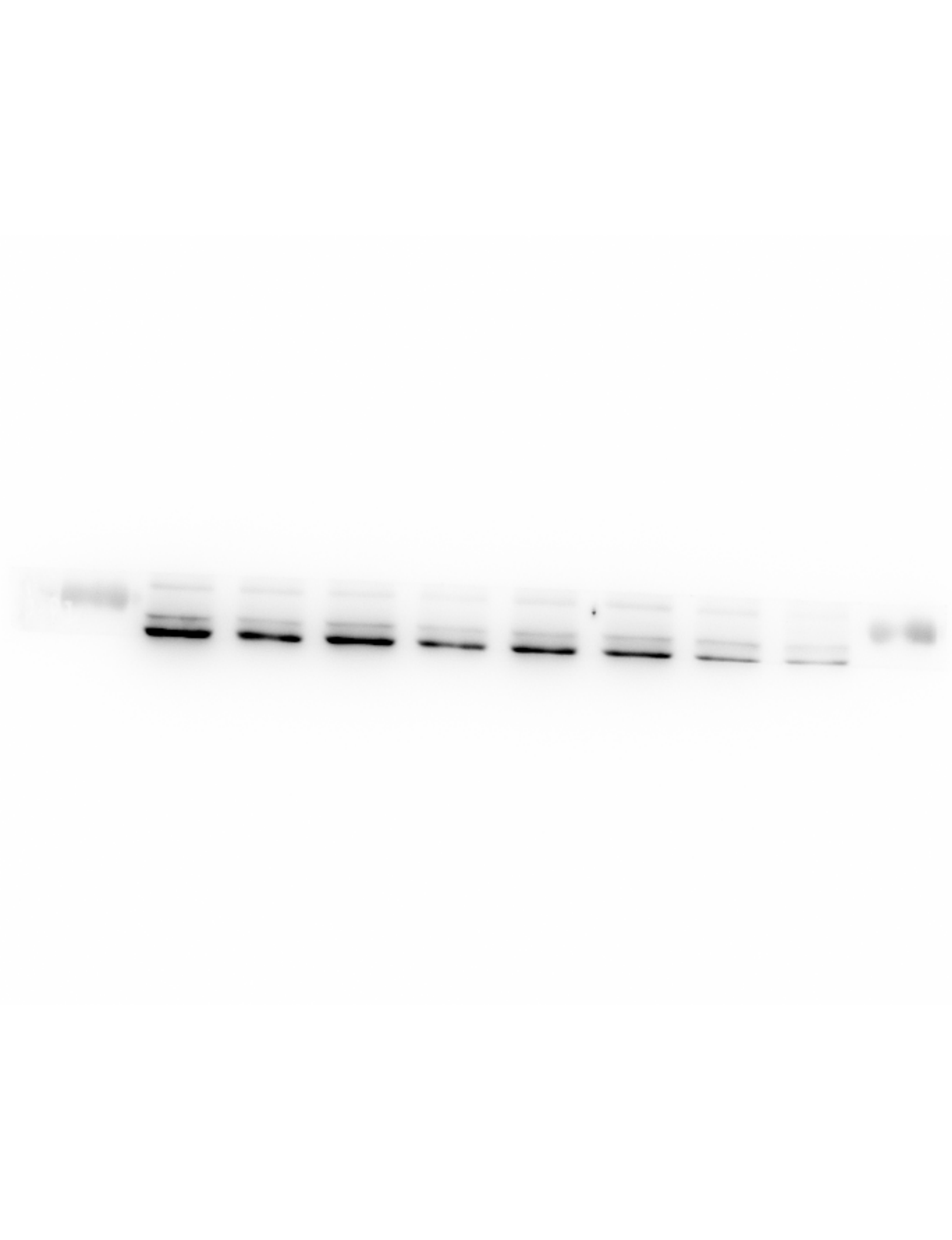

— — — — —

1 2 3 4 5

6 7

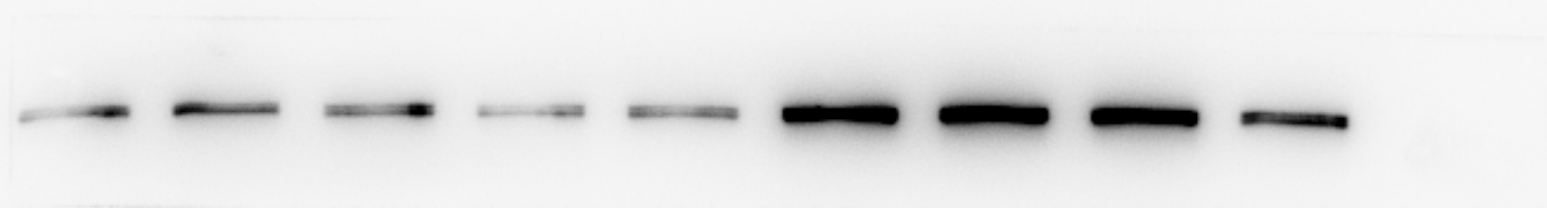

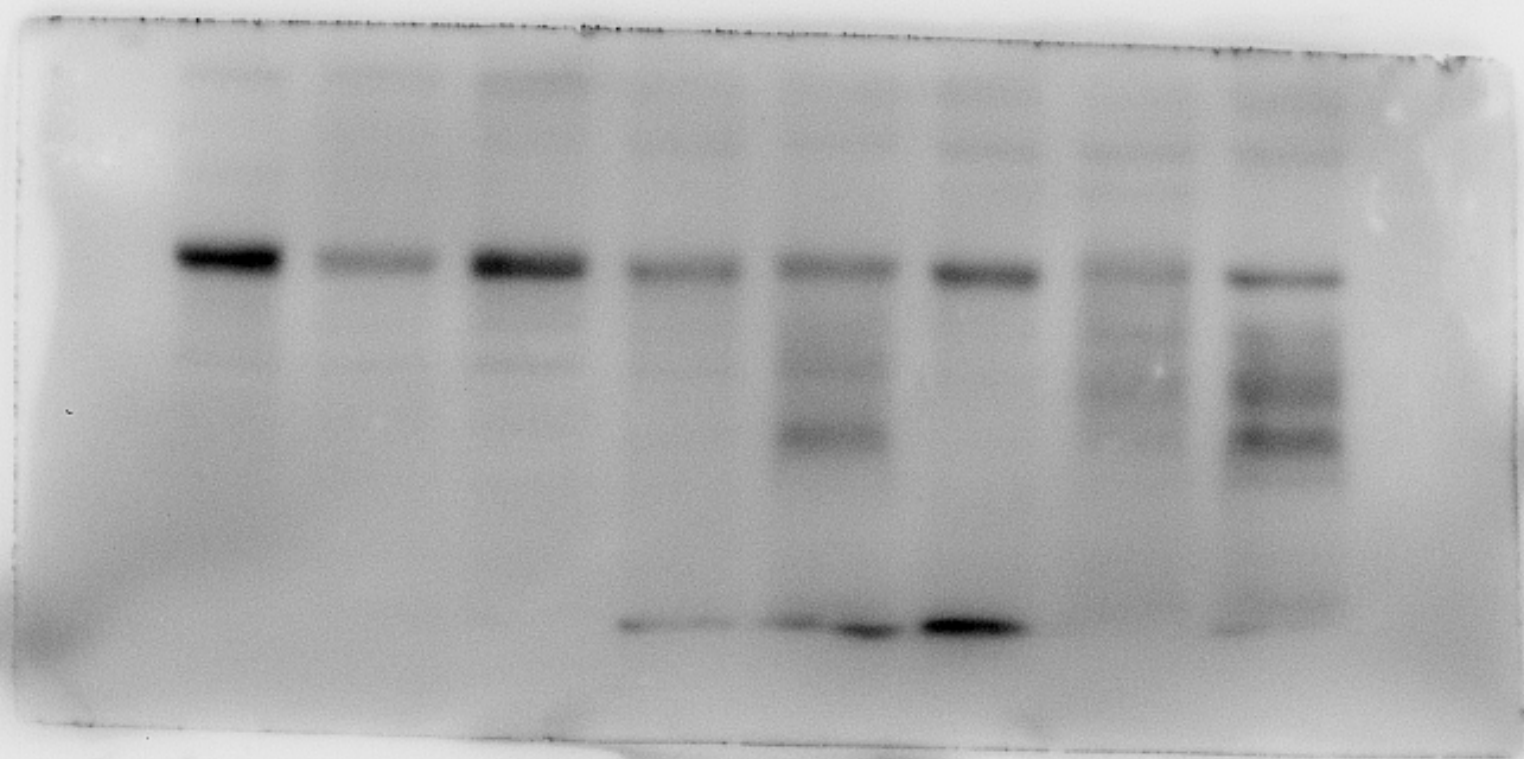

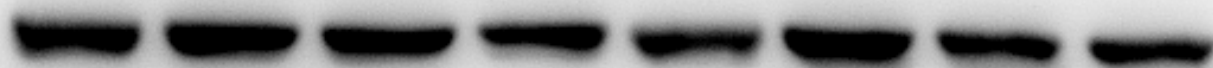

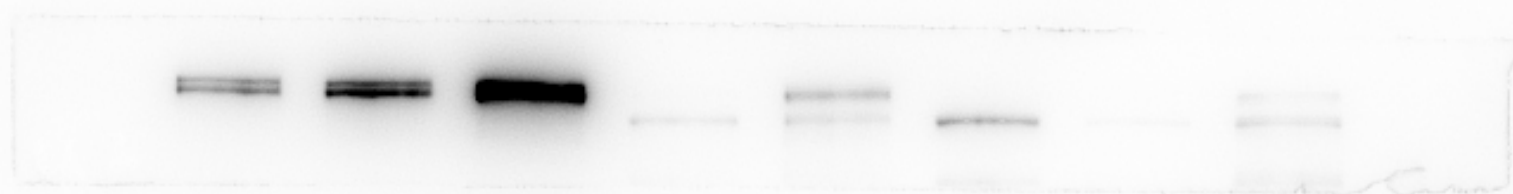

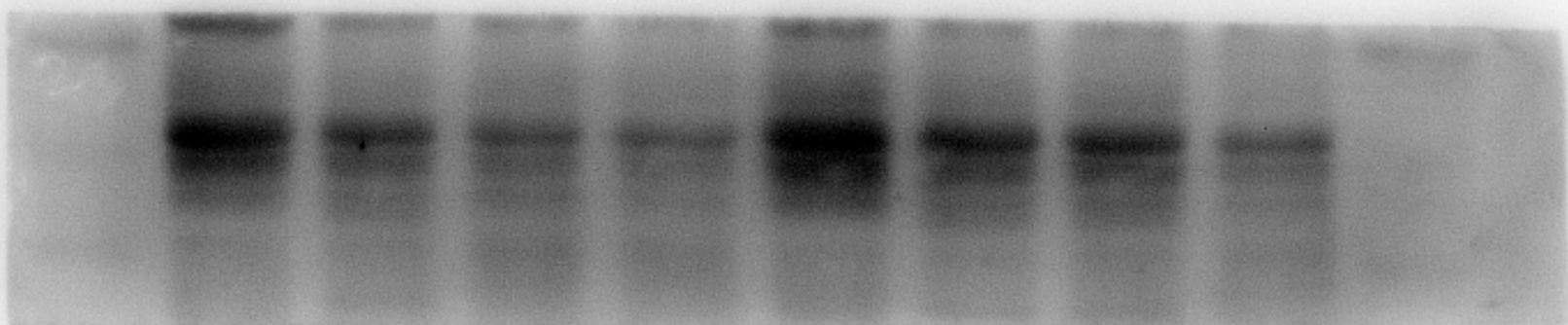

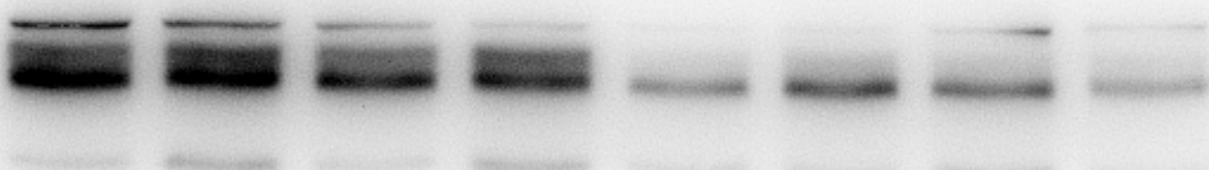

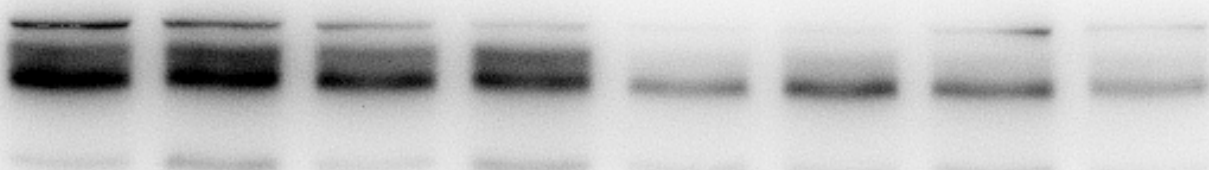

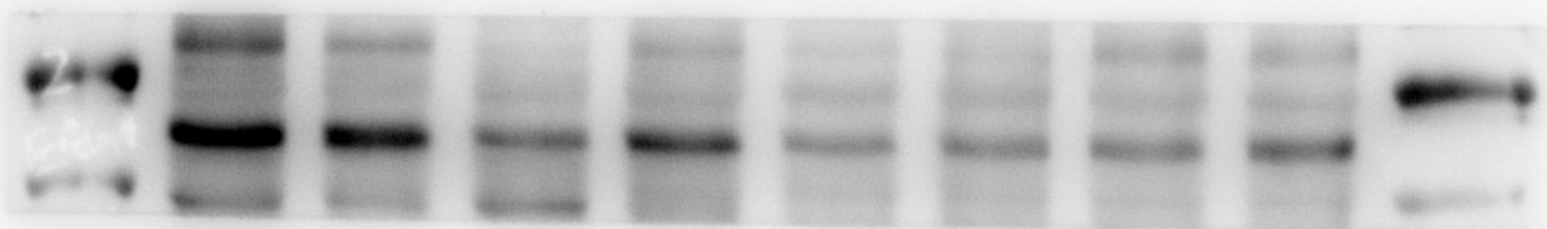

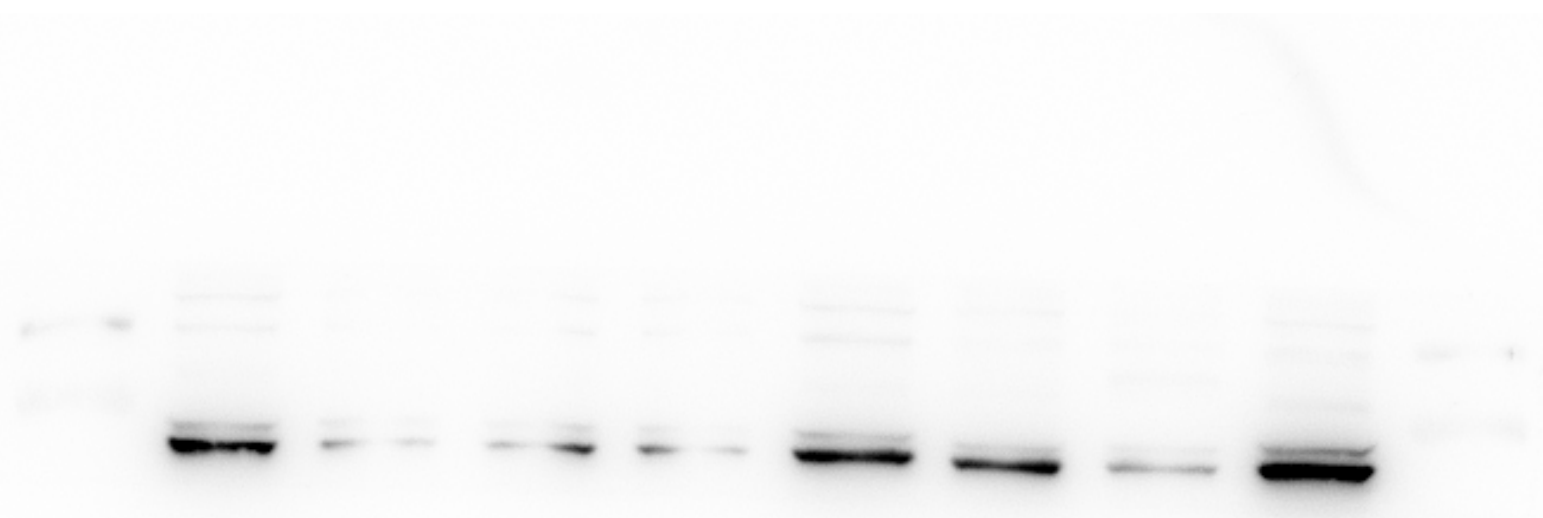

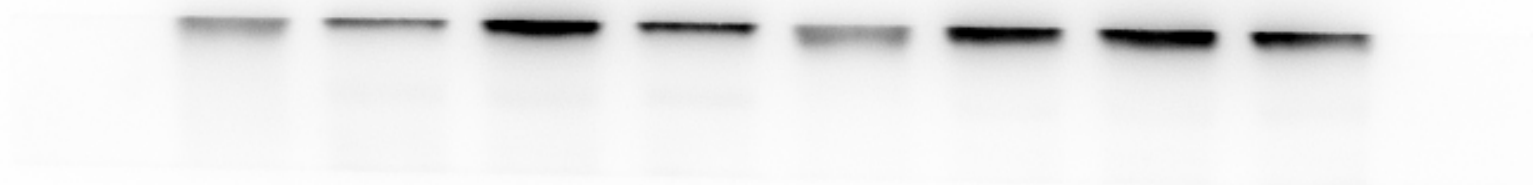

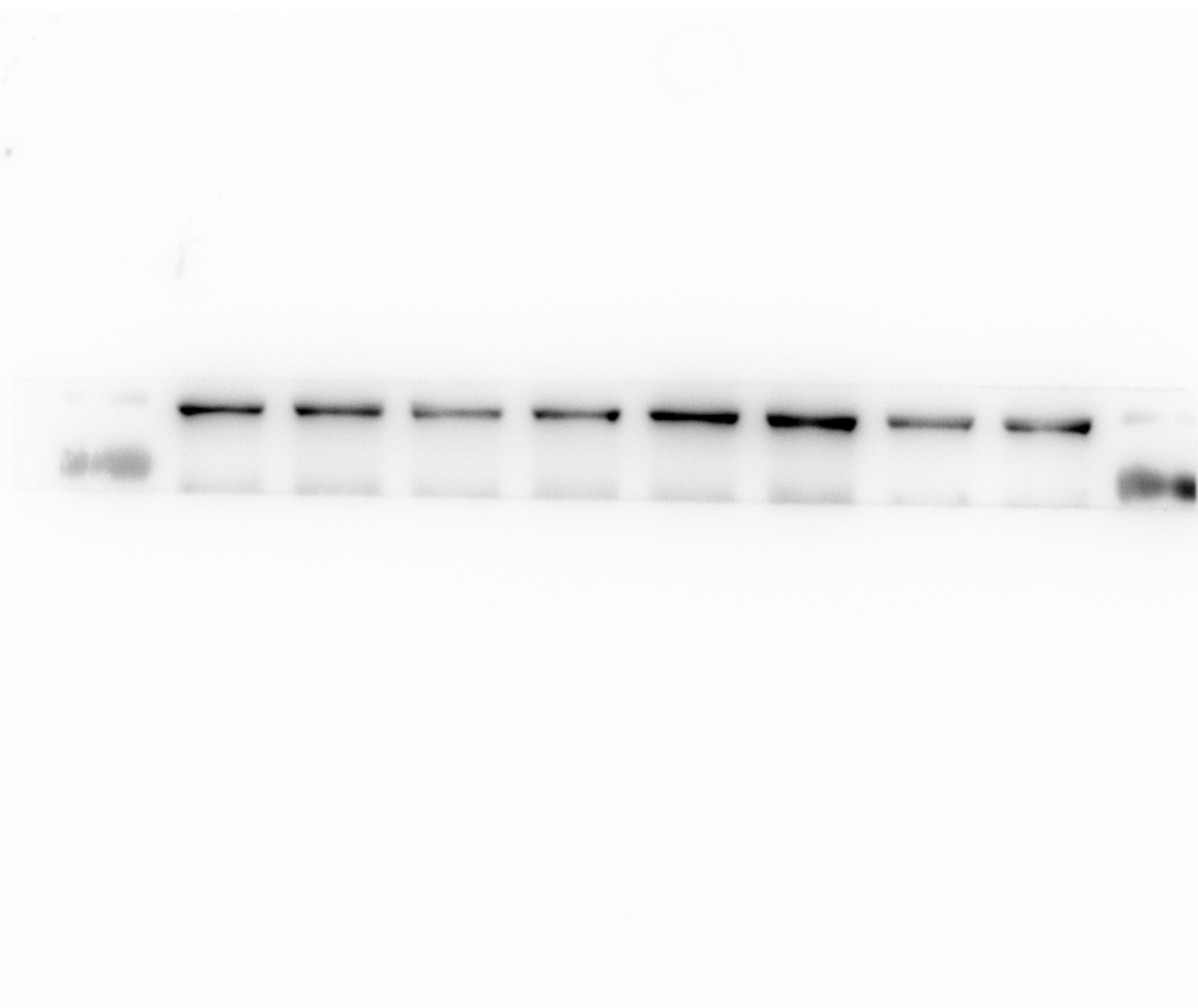

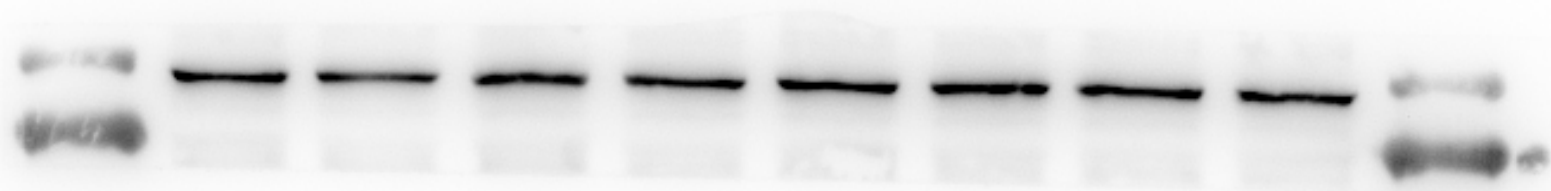

100 100 100 100 100 100 100 100

100

50

100

50

100

— — — — —

— —

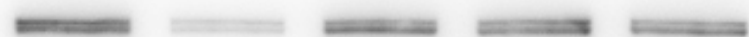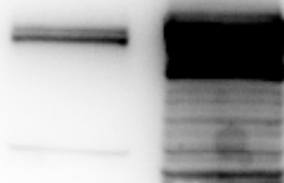

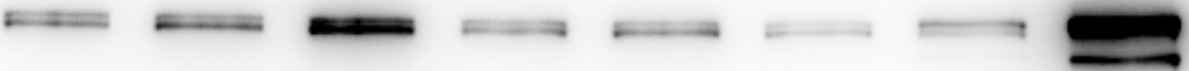

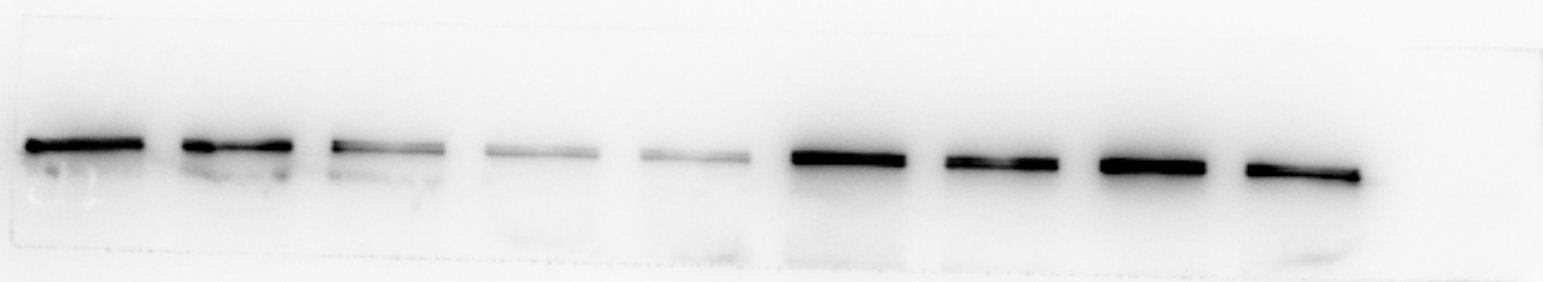

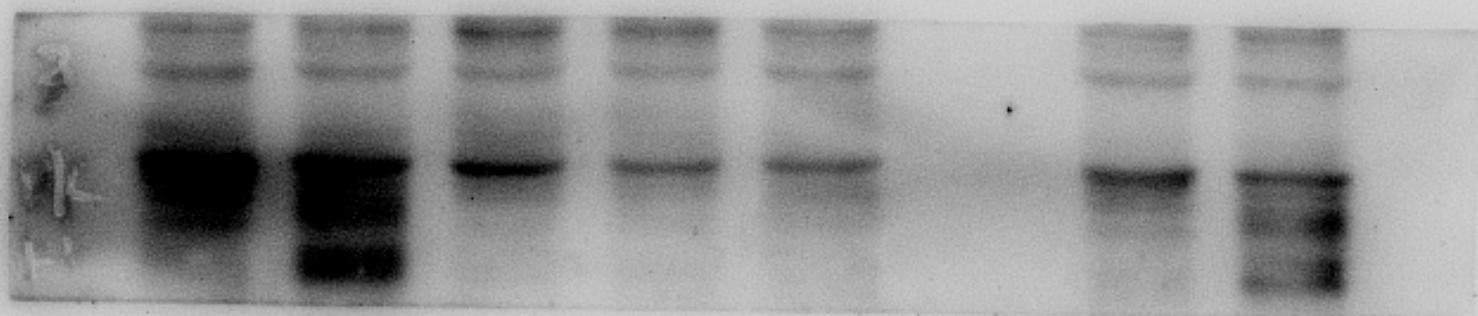

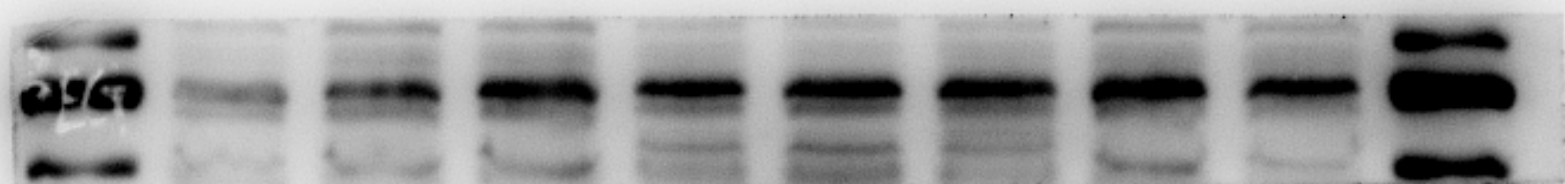

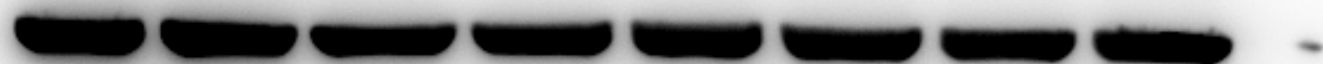

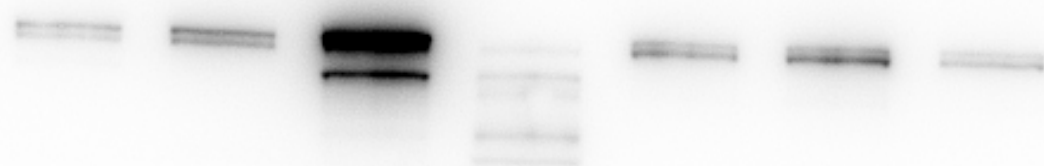

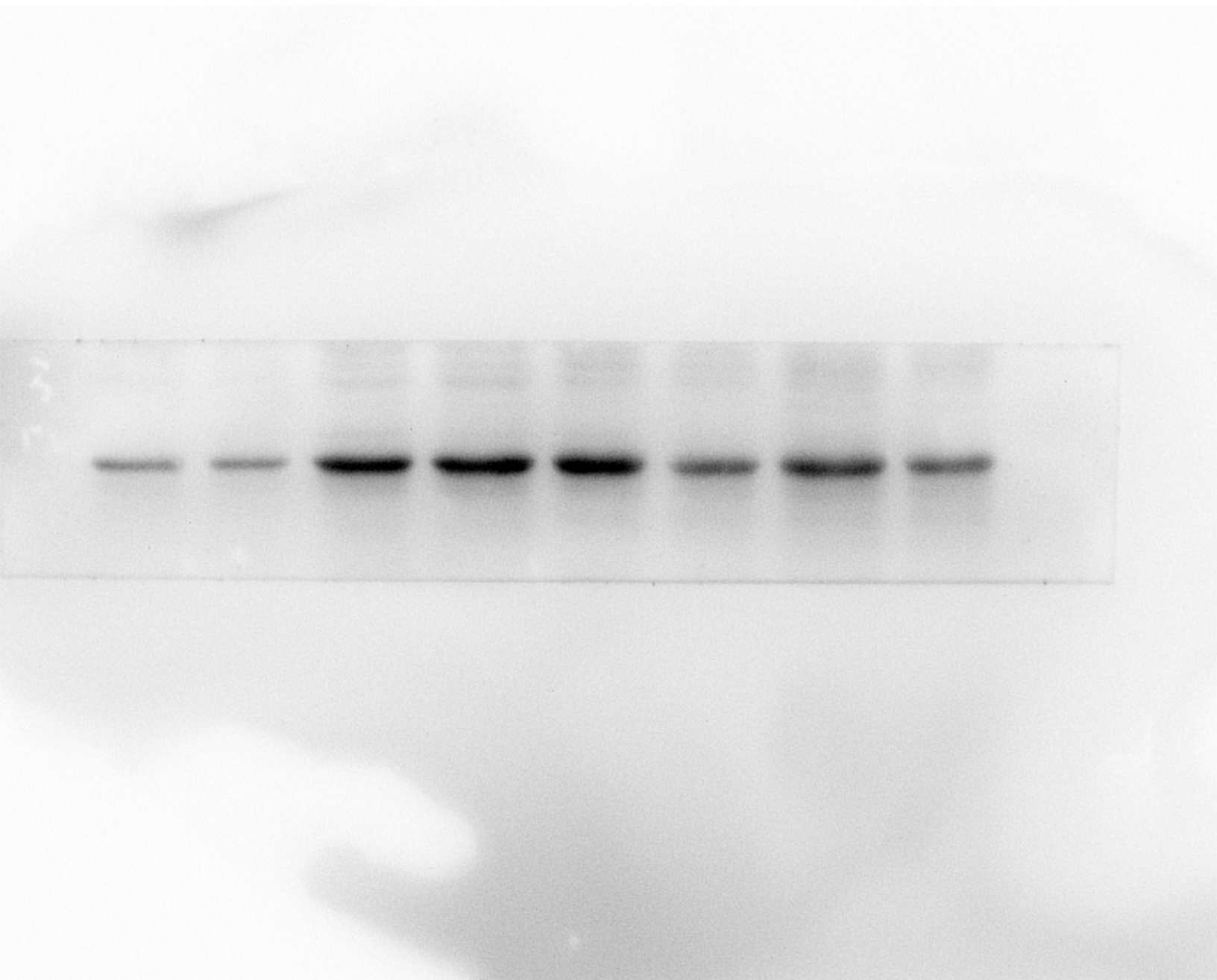

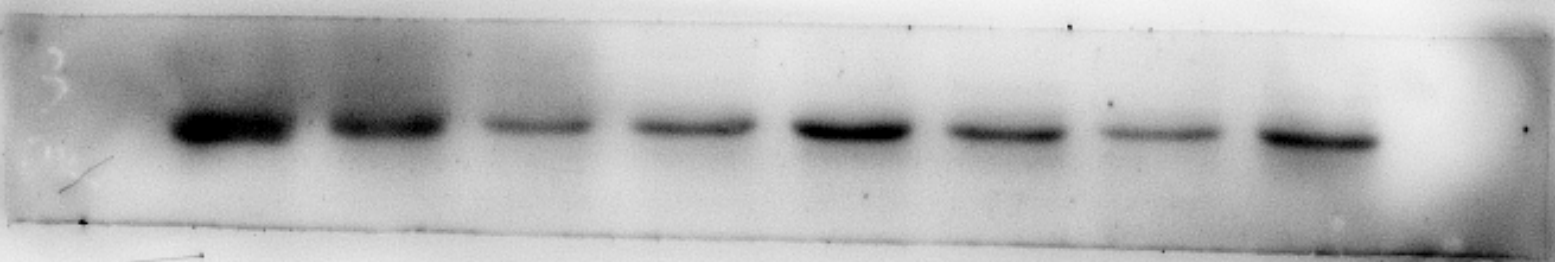

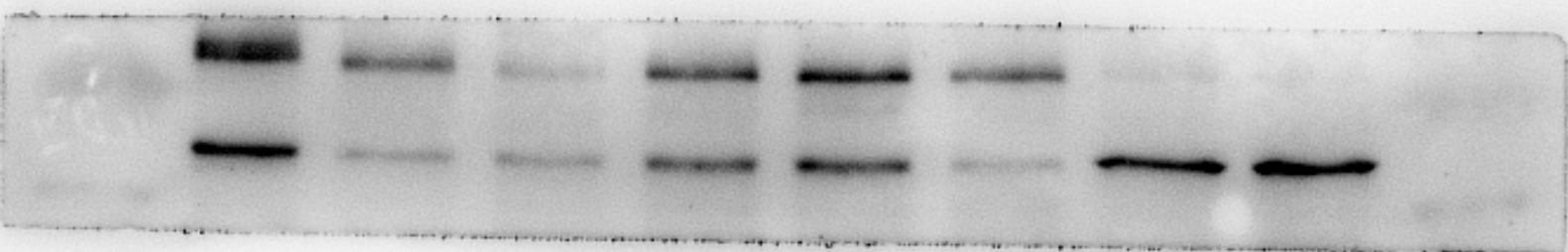

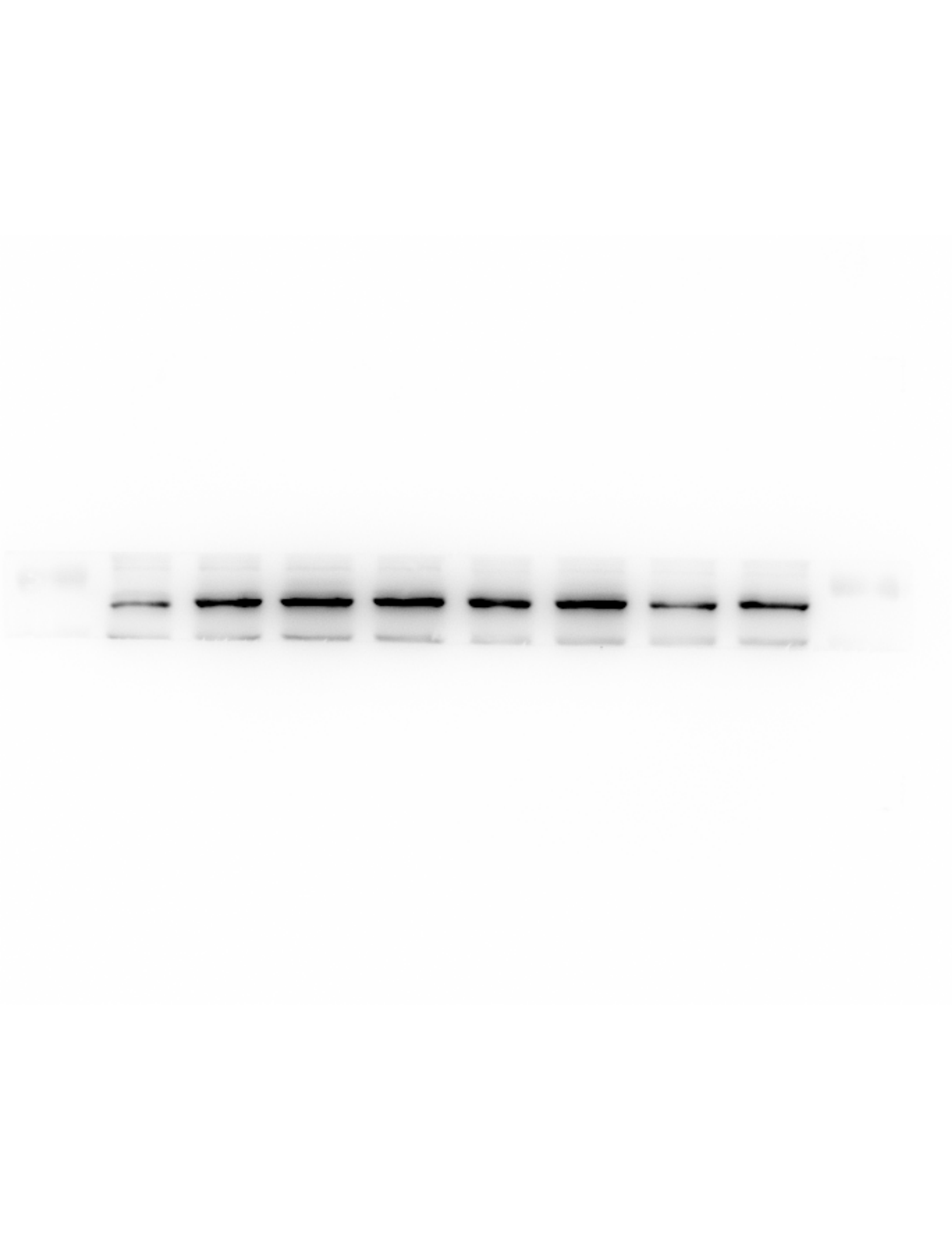

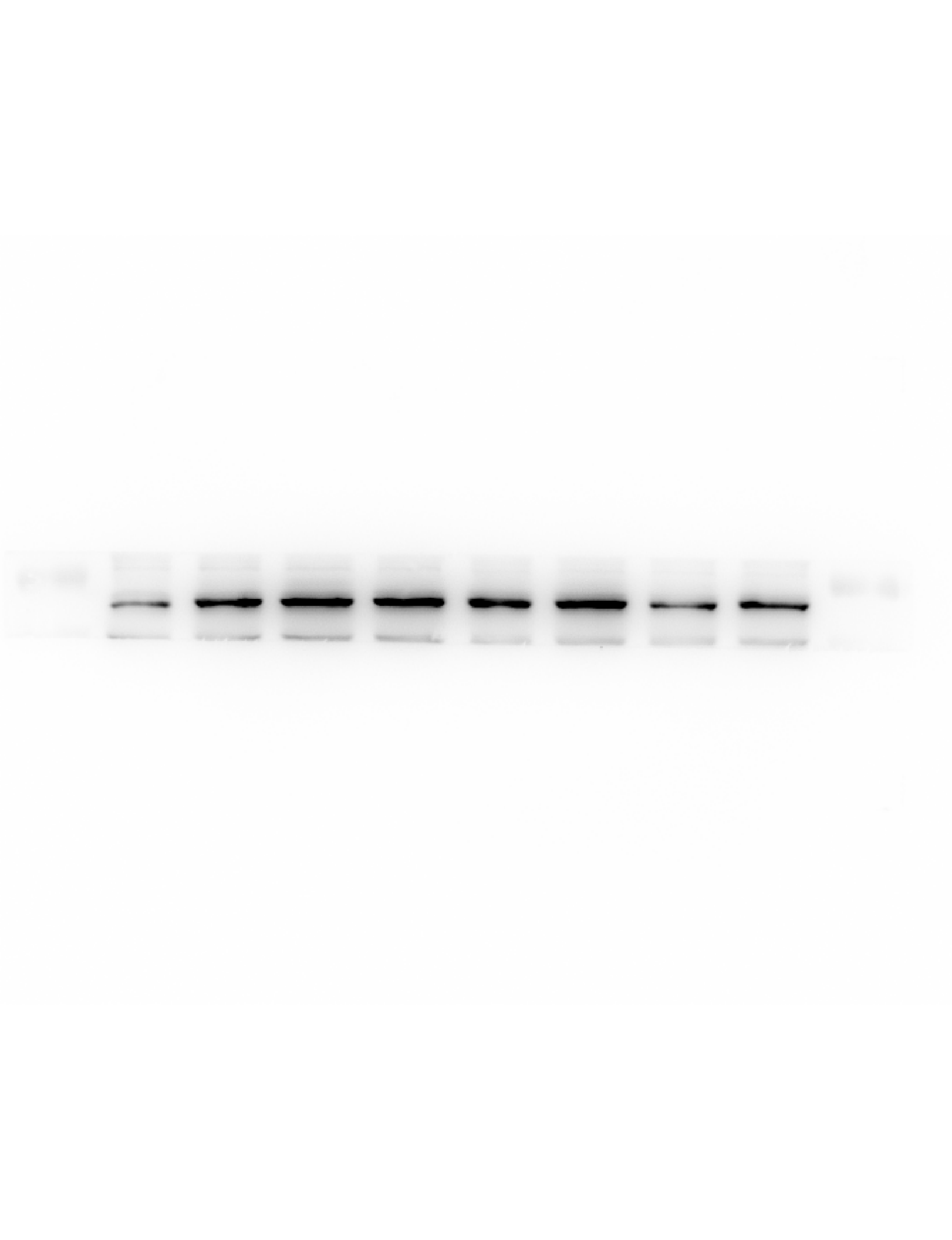

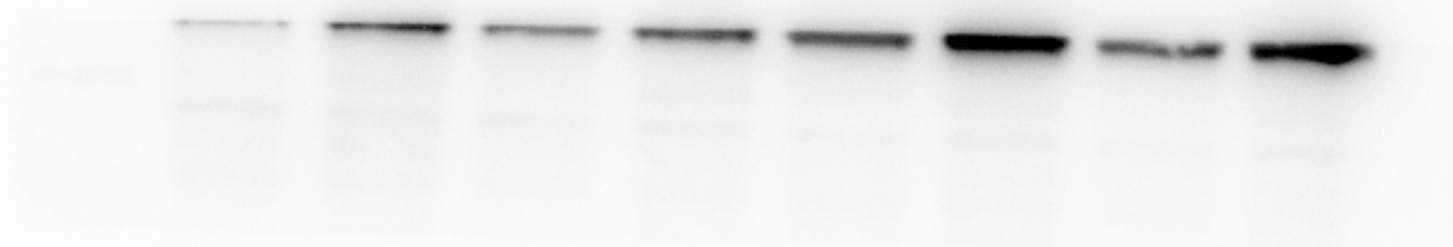

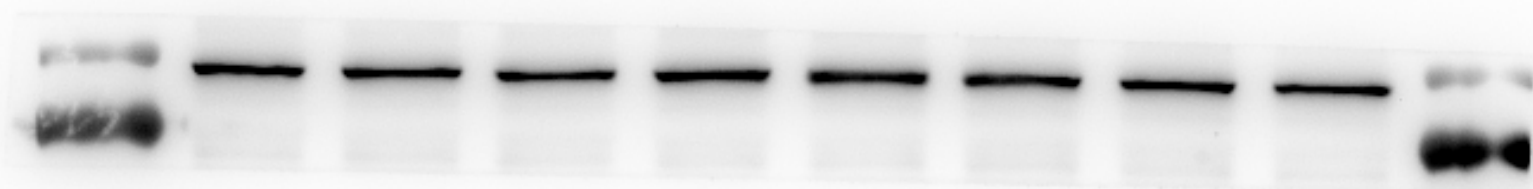

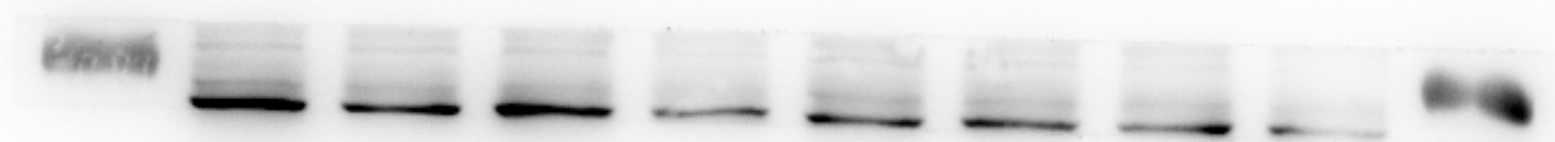

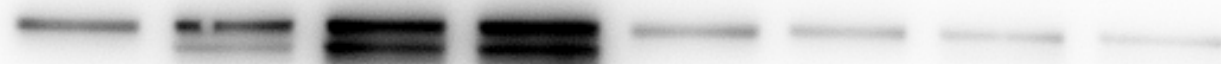

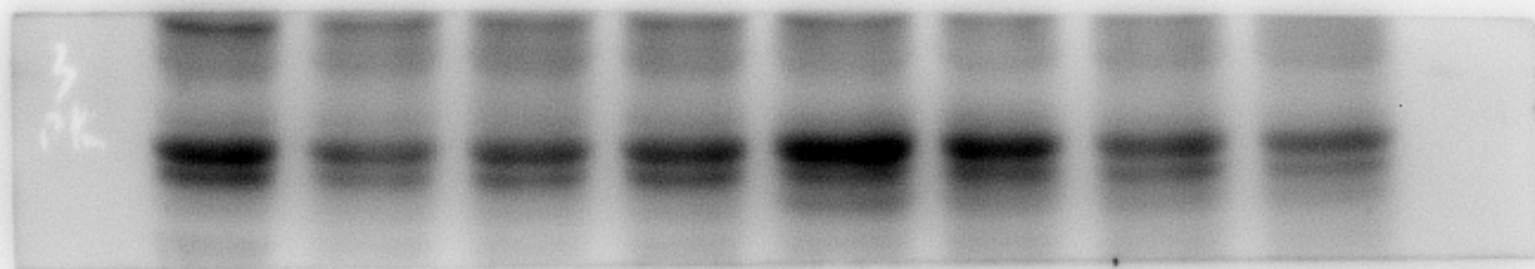

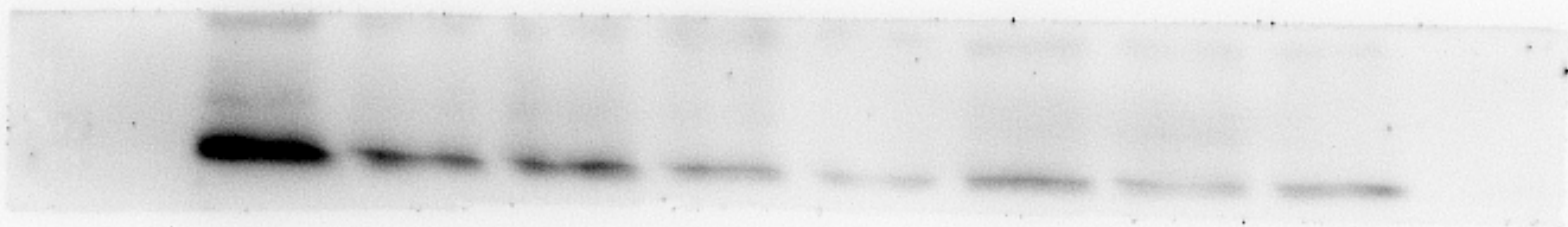

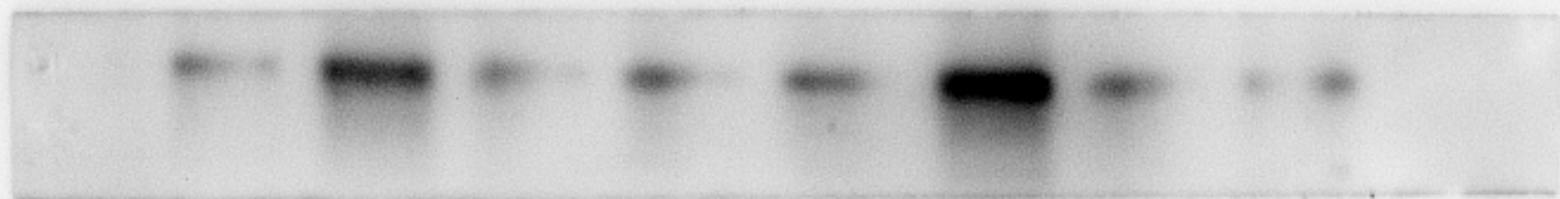

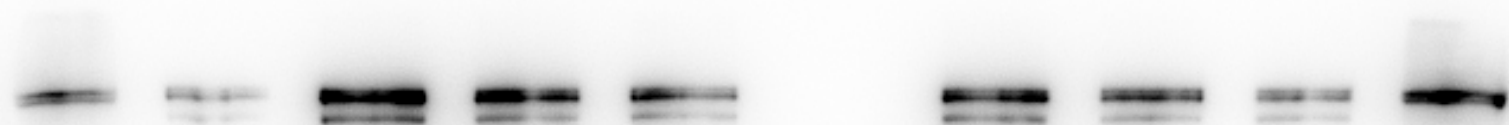

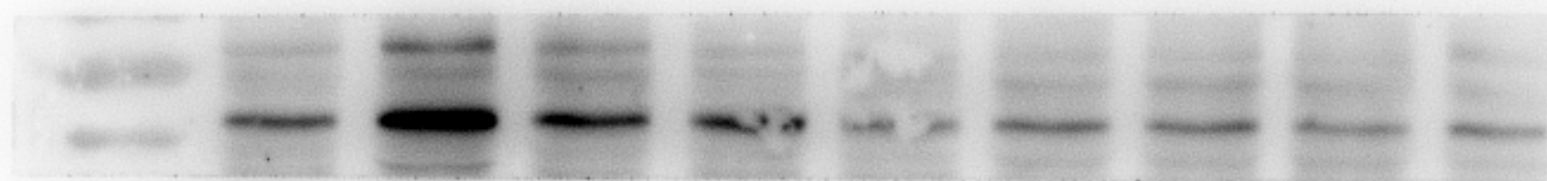

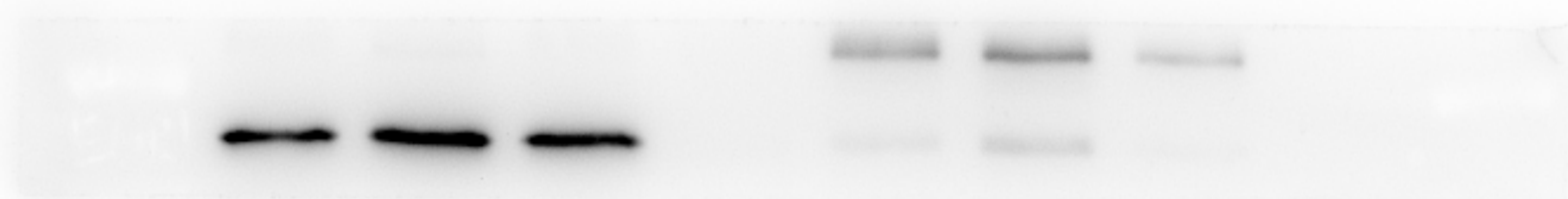

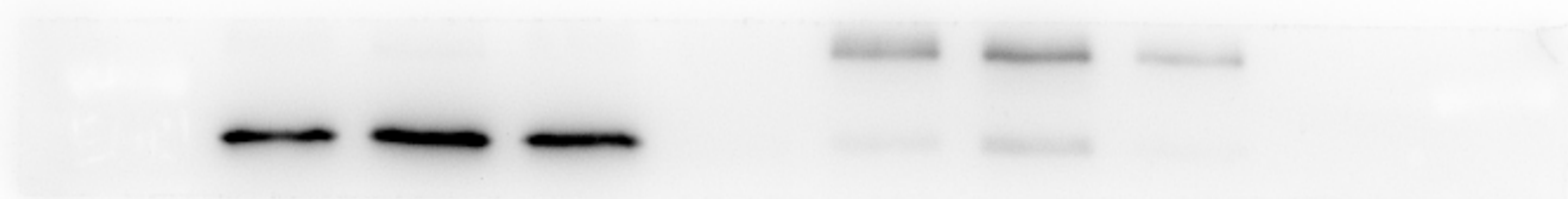

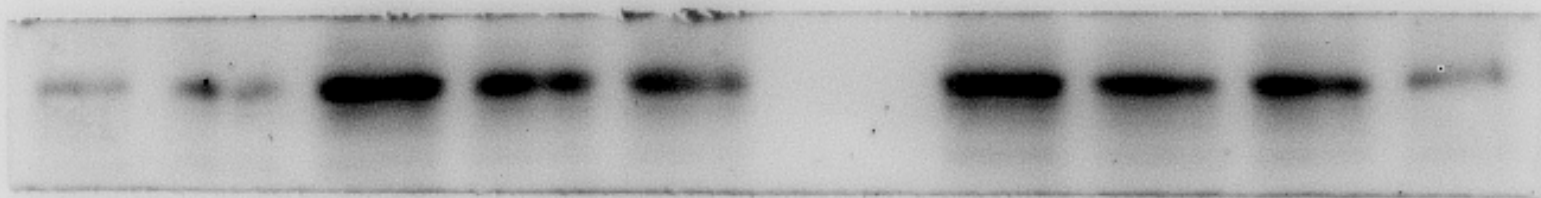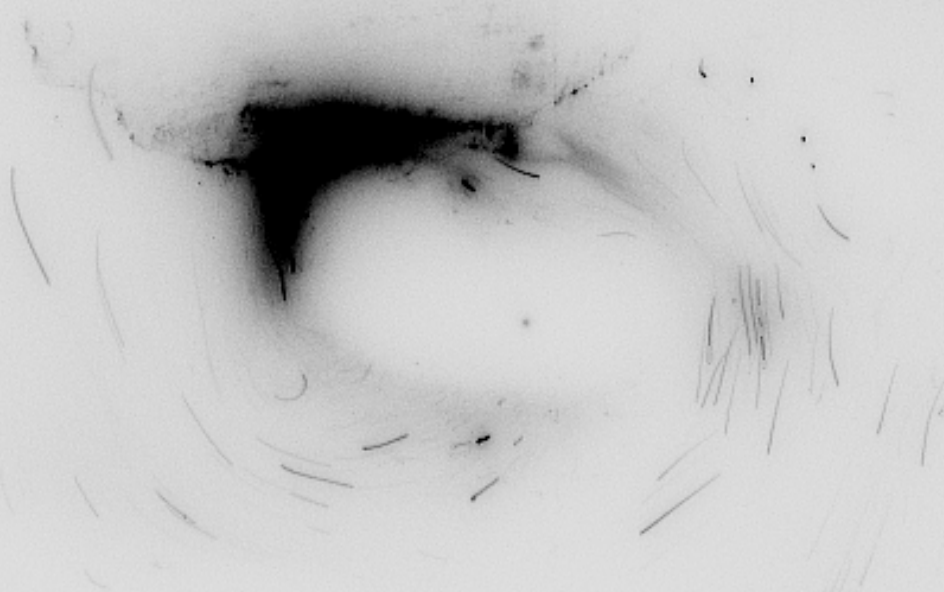

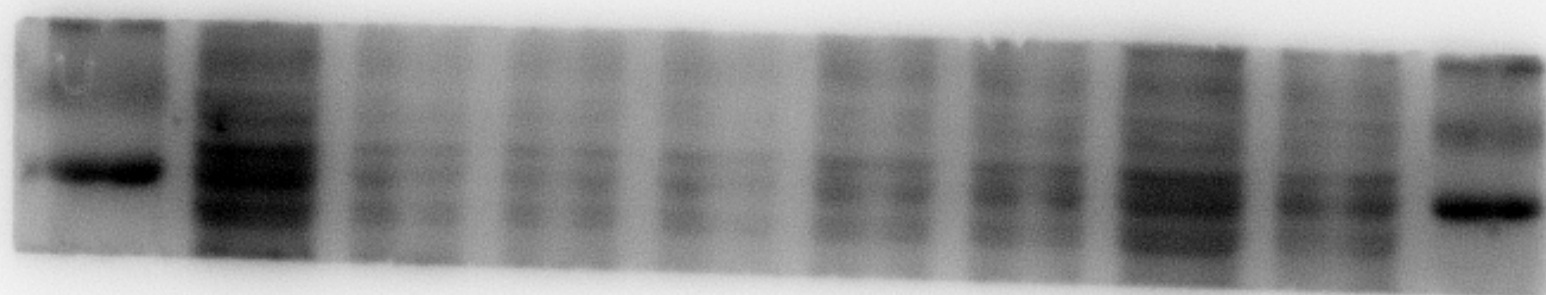

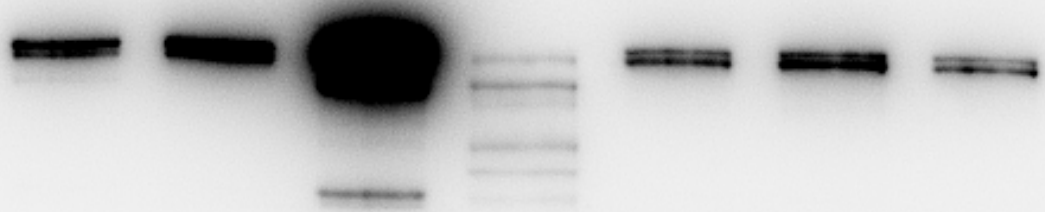

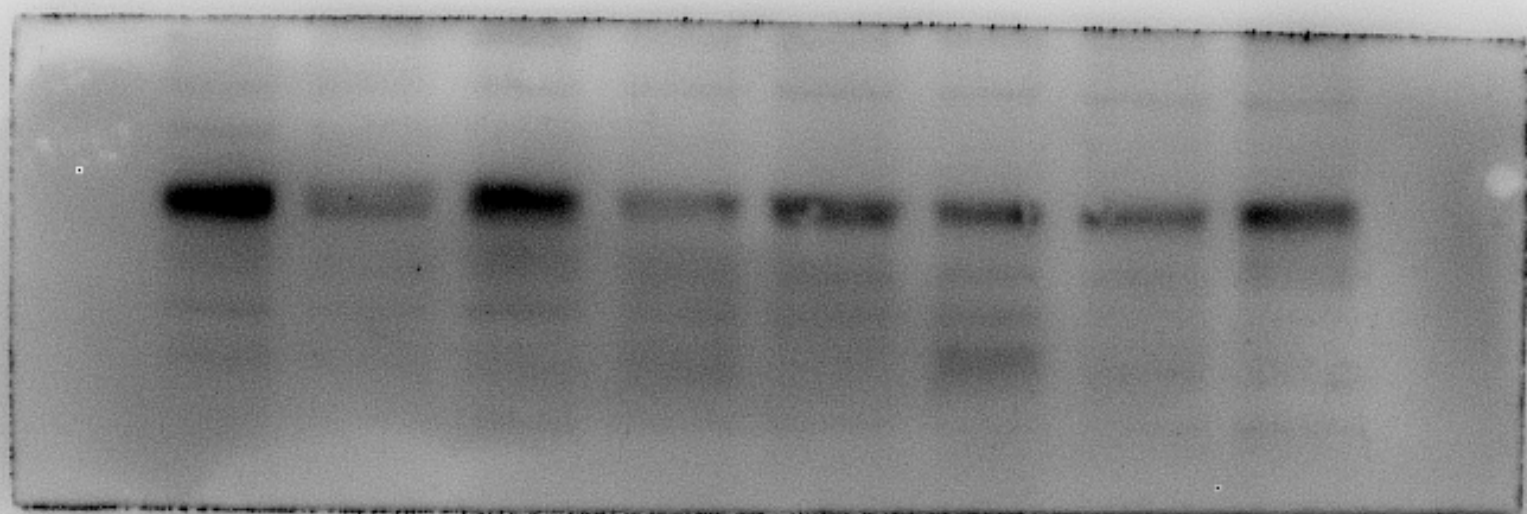

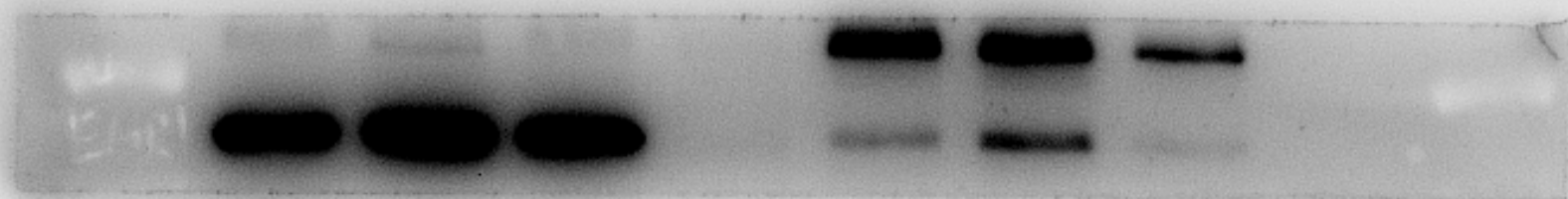

— — — — —

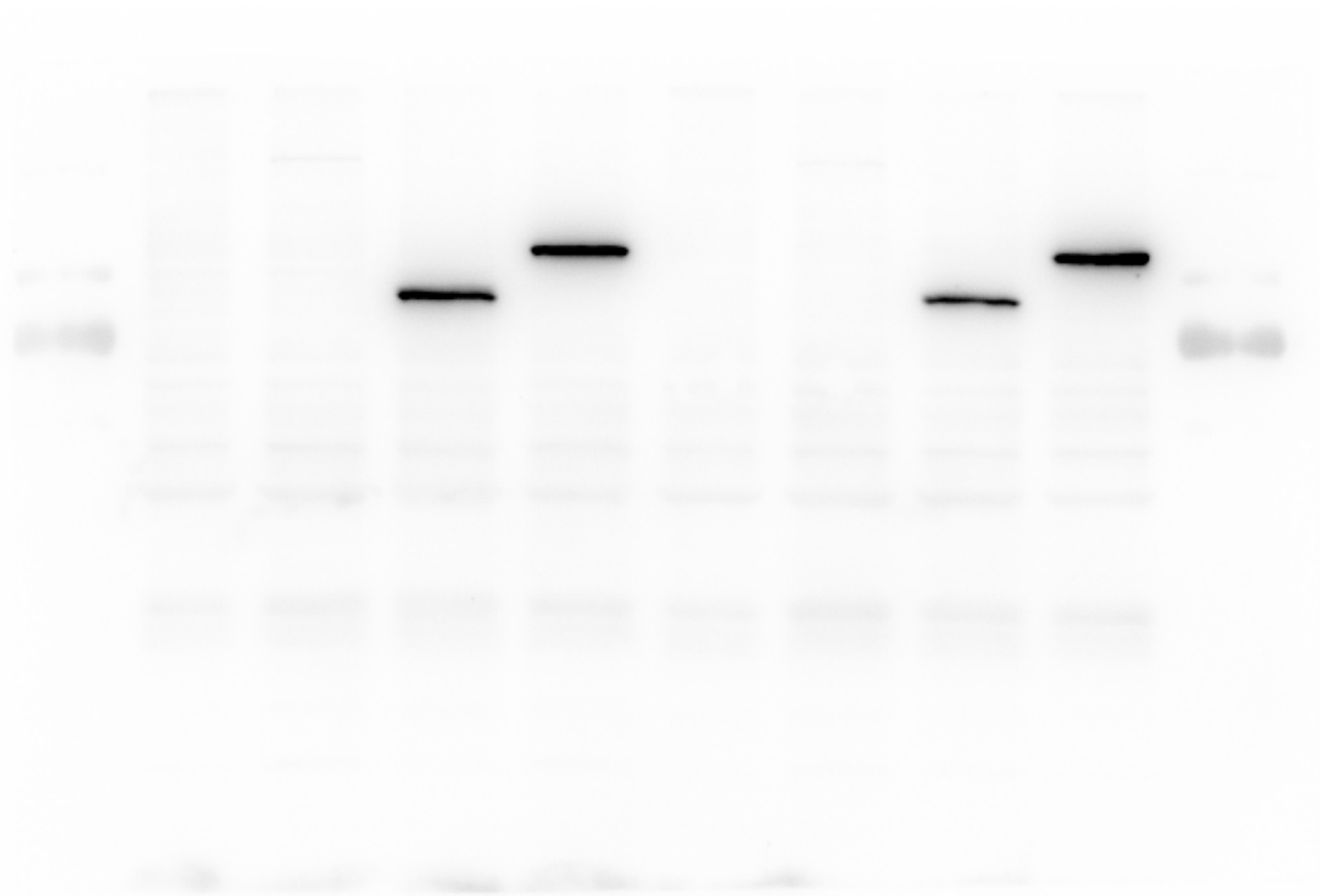

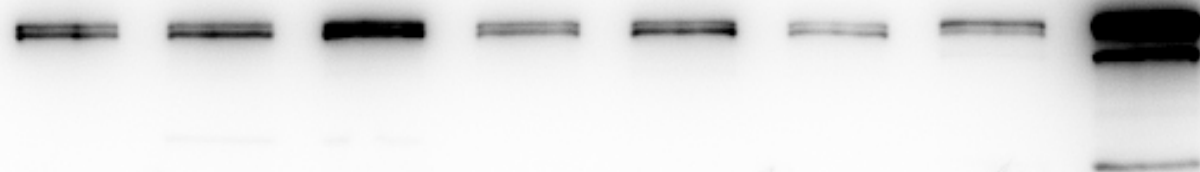

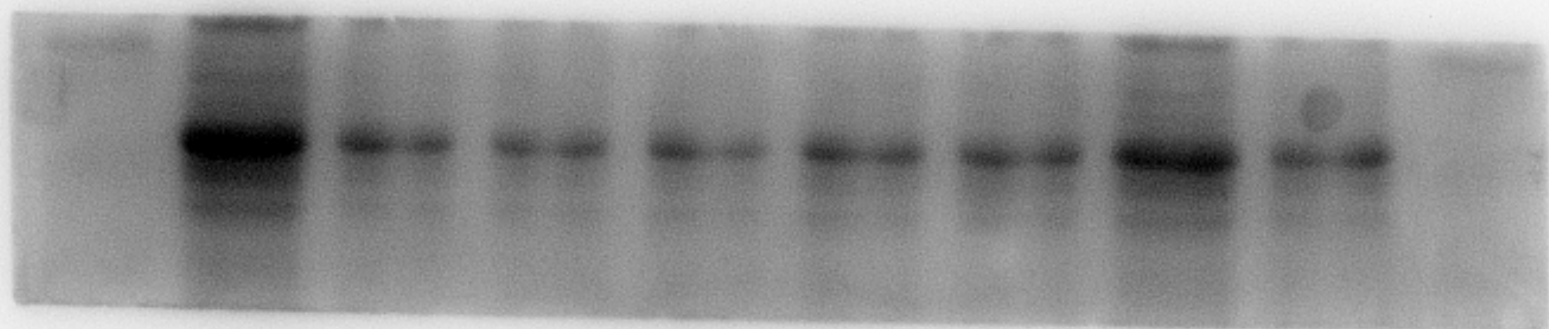

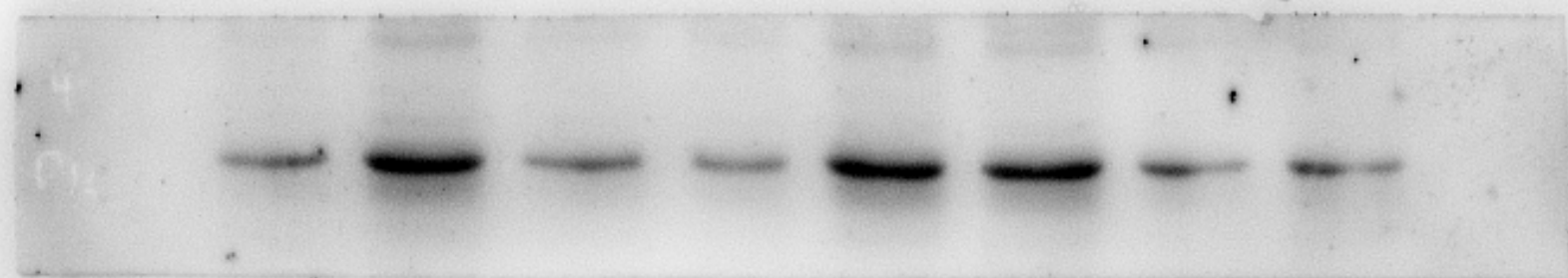

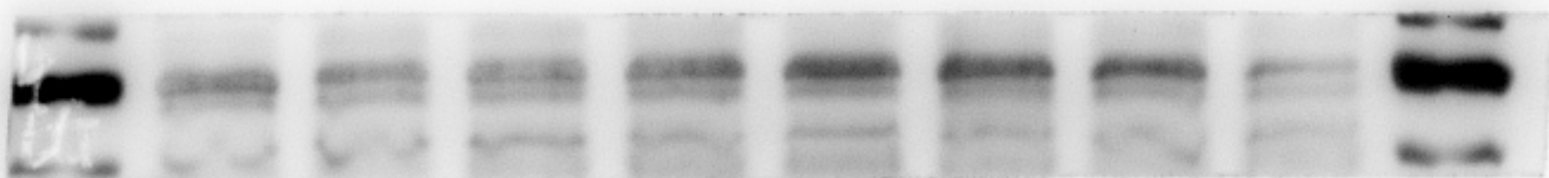

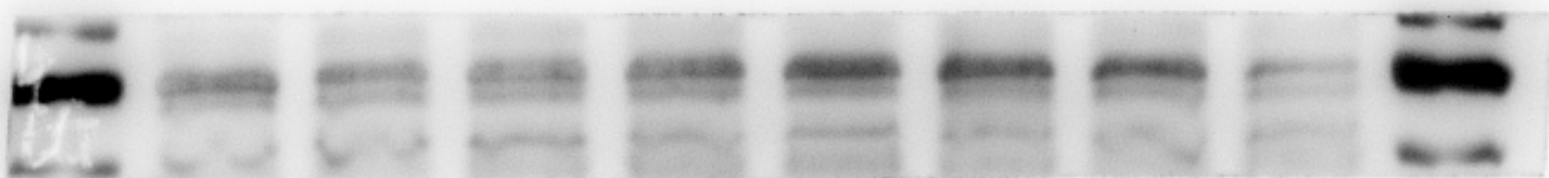

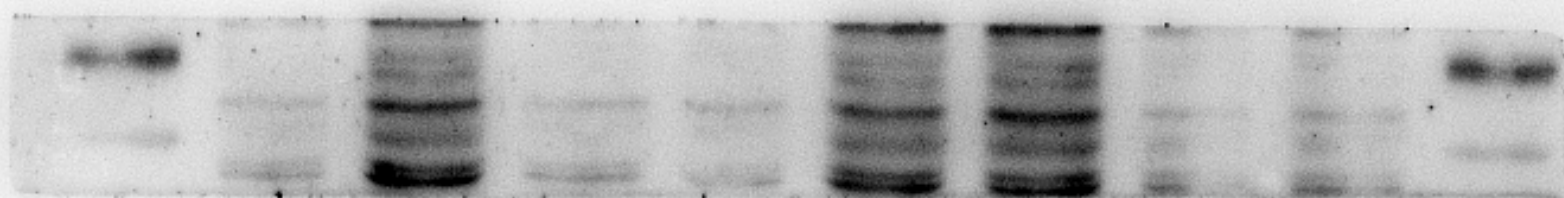

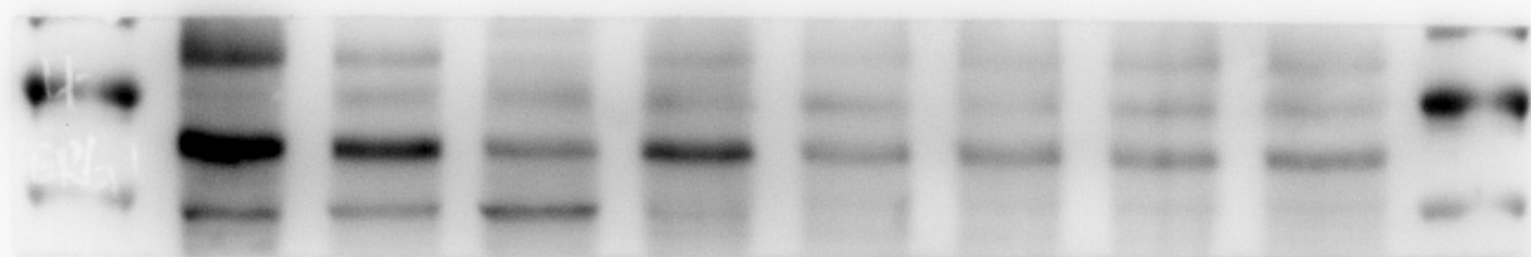

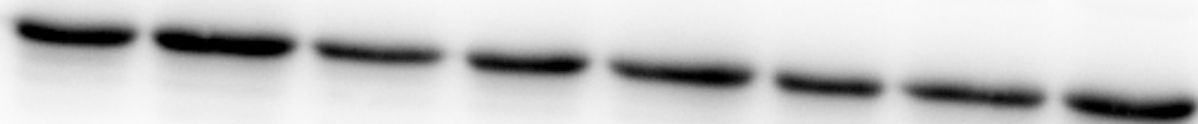

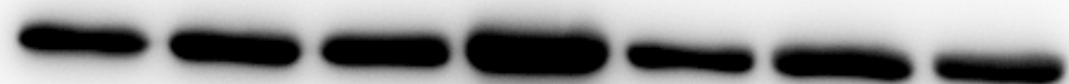

1111111

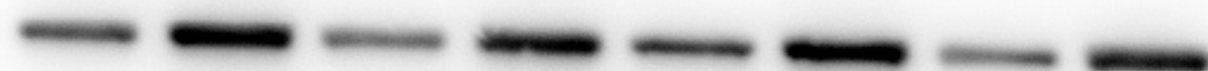

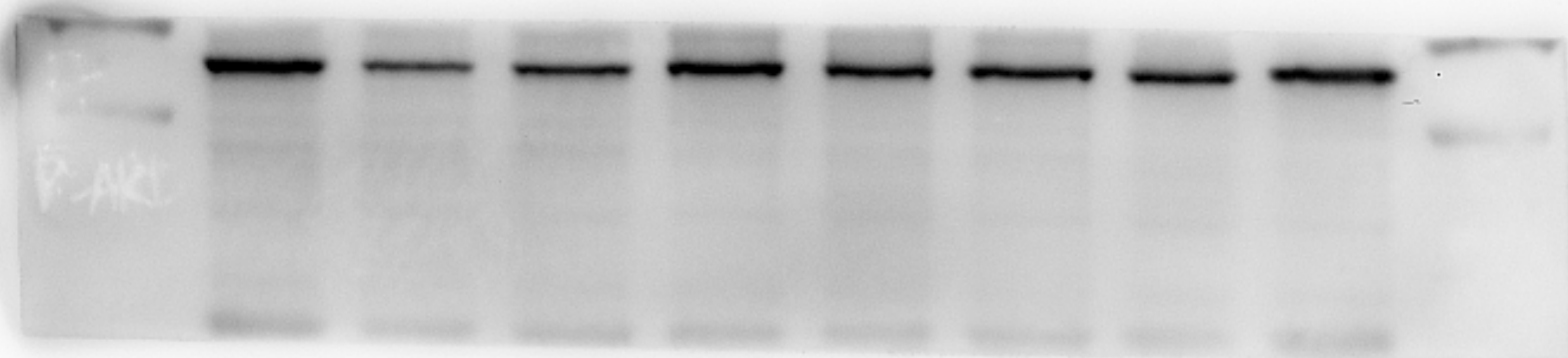

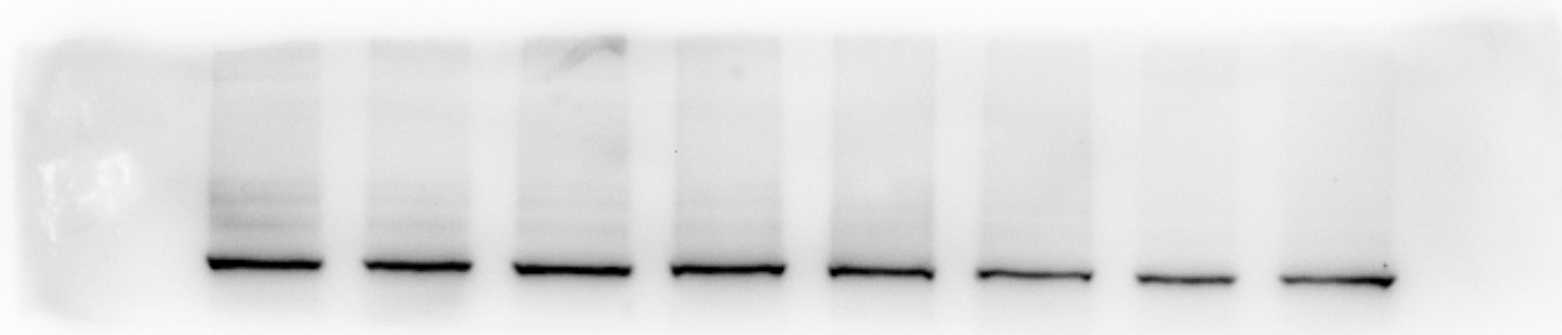

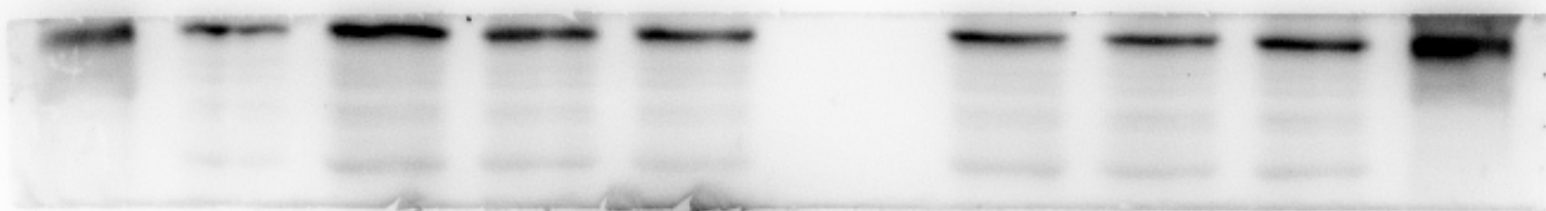

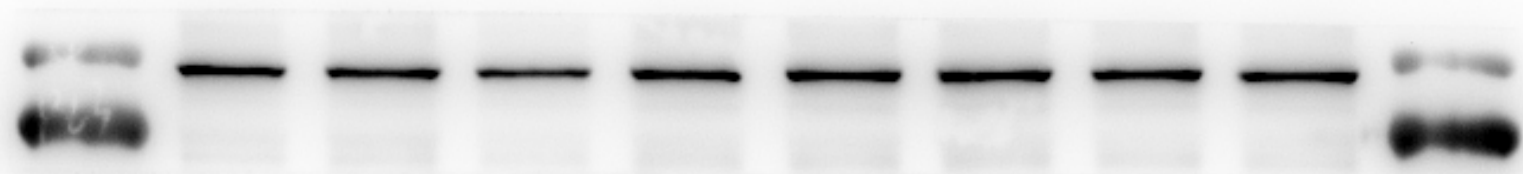

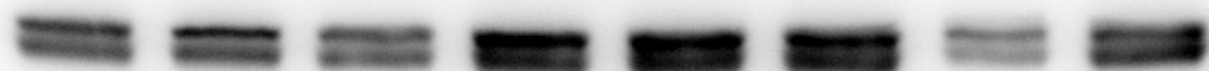

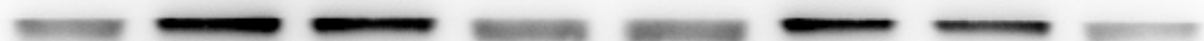

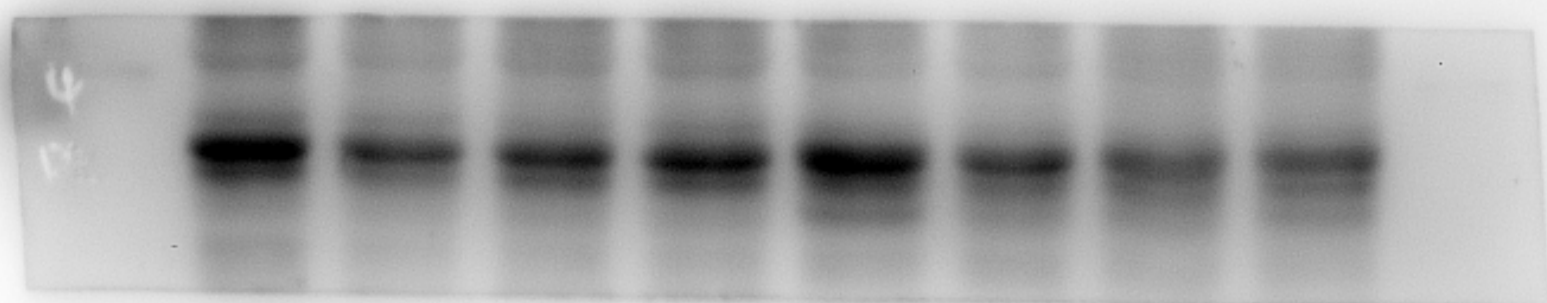

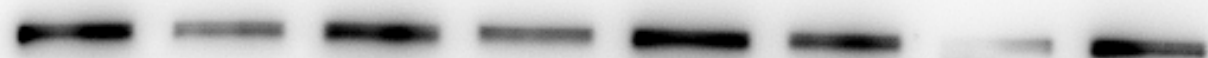

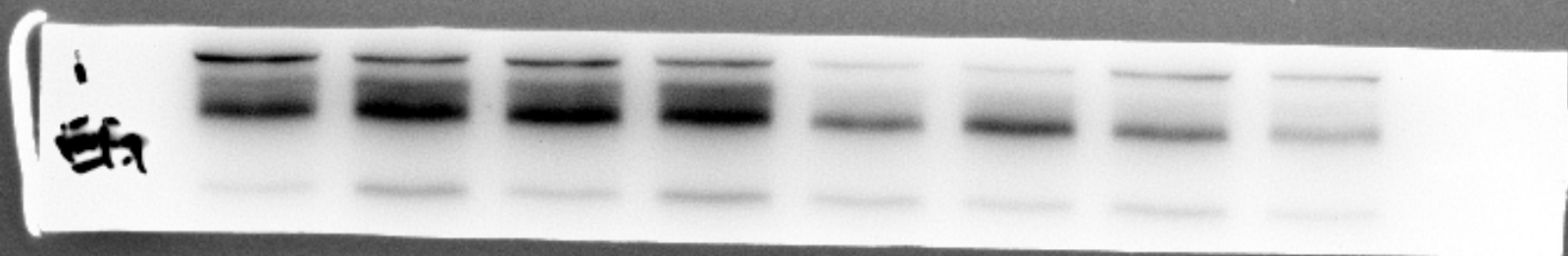

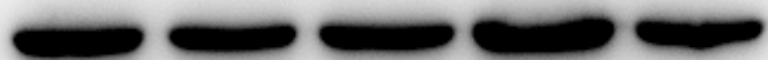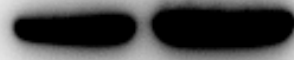

Supplement: Supplementary file 2 — Original Data File [file 41419_2022_5439_MOESM2_ESM.pdf]
